# Two *Theileria parva* CD8 T Cell Antigen Genes Are More Variable in Buffalo than Cattle Parasites, but Differ in Pattern of Sequence Diversity

**DOI:** 10.1371/journal.pone.0019015

**Published:** 2011-04-29

**Authors:** Roger Pelle, Simon P. Graham, Moses N. Njahira, Julius Osaso, Rosemary M. Saya, David O. Odongo, Philip G. Toye, Paul R. Spooner, Anthony J. Musoke, Duncan M. Mwangi, Evans L. N. Taracha, W. Ivan Morrison, William Weir, Joana C. Silva, Richard P. Bishop

**Affiliations:** 1 International Livestock Research Institute (ILRI), Nairobi, Kenya; 2 The Roslin Institute, Royal (Dick) School of Veterinary Studies, University of Edinburgh, Edinburgh, United Kingdom; 3 Institute of Comparative Medicine, Glasgow University Veterinary School, Glasgow, United Kingdom; 4 Institute for Genome Sciences and Department of Microbiology and Immunology, University of Maryland School of Medicine, Baltimore, Maryland, United States of America; Institut national de la santé et de la recherche médicale - Institut Cochin, France

## Abstract

**Background:**

*Theileria parva* causes an acute fatal disease in cattle, but infections are asymptomatic in the African buffalo (*Syncerus caffer*). Cattle can be immunized against the parasite by infection and treatment, but immunity is partially strain specific. Available data indicate that CD8^+^ T lymphocyte responses mediate protection and, recently, several parasite antigens recognised by CD8^+^ T cells have been identified. This study set out to determine the nature and extent of polymorphism in two of these antigens, Tp1 and Tp2, which contain defined CD8^+^ T-cell epitopes, and to analyse the sequences for evidence of selection.

**Methodology/Principal Findings:**

Partial sequencing of the Tp1 gene and the full-length Tp2 gene from 82 *T. parva* isolates revealed extensive polymorphism in both antigens, including the epitope-containing regions. Single nucleotide polymorphisms were detected at 51 positions (∼12%) in Tp1 and in 320 positions (∼61%) in Tp2. Together with two short indels in Tp1, these resulted in 30 and 42 protein variants of Tp1 and Tp2, respectively. Although evidence of positive selection was found for multiple amino acid residues, there was no preferential involvement of T cell epitope residues. Overall, the extent of diversity was much greater in *T. parva* isolates originating from buffalo than in isolates known to be transmissible among cattle.

**Conclusions/Significance:**

The results indicate that *T. parva* parasites maintained in cattle represent a subset of the overall *T. parva* population, which has become adapted for tick transmission between cattle. The absence of obvious enrichment for positively selected amino acid residues within defined epitopes indicates either that diversity is not predominantly driven by selection exerted by host T cells, or that such selection is not detectable by the methods employed due to unidentified epitopes elsewhere in the antigens. Further functional studies are required to address this latter point.

## Introduction

*Theileria parva* is a tick-transmitted protozoan parasite that causes East Coast fever (ECF), an economically important disease of cattle in eastern, central and southern Africa. The life cycle in the bovine host involves two intracellular stages: the schizont, which transforms lymphocytes and is responsible for disease pathology, and the intra-erythrocytic piroplasm, which is infective for the tick vector, *Rhipicephalus appendiculatus* [1]. The mammalian life cycle stages are haploid, but there is a transient diploid phase in the tick gut after fusion of gametes [2] and sexual recombination has been observed between and within *T. parva* stocks during experimental transmission [3], [4], [5]. The African buffalo (*Syncerus caffer*) is the natural reservoir of *T. parva*, but the parasite does not cause disease in this species. Transmission of buffalo-derived *T. parva* to cattle results in a rapidly lethal disease, but in many cases the parasites do not differentiate to the erythrocyte-infective stage and are not transmissible by ticks [6], [7]. Hence, although *T. parva* parasites that originate from buffalo are genotypically and antigenically closely related to *T. parva* maintained in cattle, available evidence indicates that a significant proportion of the buffalo-derived population cannot be transmitted between cattle.

Immunisation of cattle by infection with *T. parva* and simultaneous treatment with long-acting tetracycline results in long term immunity against the homologous parasite strain but variable protection against challenge with heterologous parasite strains. Hence, vaccination of cattle in the field by this method requires a mixture of parasite strains. Studies of immune responses in cattle immunised by infection and treatment have provided evidence that MHC-I restricted CD8^+^ T cells, which are able to kill parasitised lymphoblasts, are important mediators of immunity [8], [9]. CD8^+^ T-cell responses in cattle immunized with a single parasite isolate frequently exhibit parasite strain specificity [9], [10] and in one study such strain specificity has been shown to correlate with immune status upon challenge with a heterologous strain [11]. These findings suggest that during parasite evolution there may have been immune-imposed selection for sequence diversity in the target antigens of the protective immune response.

Identification of molecular diversity in *T. parva* was initially based on application of a panel of monoclonal antibodies, mainly directed against a single immunodominant polymorphic antigen [12]. This was subsequently combined with detection of restriction fragment length polymorphisms in genomic DNA using probes derived from multi-copy gene families (reviewed in [4]). The recently sequenced *T. parva* genome has been used to identify a panel of DNA satellite markers allowing more comprehensive genotyping of parasite isolates [13]. Population studies of *T. parva* from different regions of Uganda and Kenya, using a subset of these markers, revealed a high level of diversity and a high frequency of infection of cattle with mixed genotypes [14], [15]. Although some evidence of geographical sub-structuring was found among parasite populations, the lack of linkage disequilibrium between alleles at different loci was consistent with frequent genetic exchange.

Although these genotypic analyses have provided insight into the genetic structure of *T. parva* populations, they are uninformative with respect to the nature and selective pressures driving antigenic diversity relevant to immune protection. The recent identification of a number of *T. parva* antigens and epitopes recognised by CD8^+^ T cells from *T. parva*–immune cattle [16], [17] now provides an opportunity to address this question. Detailed studies of immune responses to two of these antigens, Tp1 and Tp2, have demonstrated that they are highly dominant targets of the CD8^+^ T-cell response in cattle expressing the A18 and A10 class I MHC haplotypes, respectively. Preliminary analyses of Tp1 and Tp2 sequences in a small number of laboratory parasite strains revealed that the epitopes recognised by specific CD8^+^ T cells are polymorphic and that this is associated with differential recognition by CD8^+^ T cells [18].

The present study set out to determine the extent and nature of sequence diversity in genes encoding the Tp1 and Tp2 antigens in a series of isolates of *T. parva* obtained from cattle and buffalo in regions of Africa where ECF is endemic, and to interrogate the sequence data for evidence of the mode and strength of selection.

## Materials and Methods

### Ethics statement

The ILRI's Institutional Animal Care and Use Committee (IACUC) was established in 1993 to ensure that international standards for animal care and use are followed in all ILRI research involving use of animal. ILRI has complied voluntarily with the UK's Animals (Scientific Procedures) Act 1986 (http://www.homeoffice.gov.uk/science-research/animal-research/) that contains guidelines and codes of practice for the housing and care of animals used in scientific procedures. The study reported here was carried out in strict accordance with the recommendations in the standard operating procedures of the ILRI IACUC and adequate consideration of the 3R's (Replacement of animal with non-animal techniques, Reduction in the number of animals used, and Refinement of techniques and procedures that reduce pain and distress). The ILRI' Experimental Animal Request Form and Protocol for lymph node biopsies and blood collection was approved by the ILRI IACUC (IACUC ref no. 2006.9, IACUC ref 2006.10 and IACUC ref 2007.10).

### Parasite isolates

The 82 *T. parva* schizont-infected cell lines used in this study were established and maintained using previously described methodologies [19]. These comprised lines derived from four different sources (Table S1).

#### (i) Laboratory samples (LS)

Nine cell lines were generated by infection of bovine lymphocytes *in vitro* with sporozoite stabilates of established laboratory isolates [19], [20] while eight were established from biopsies from animals infected with these stabilates (Table S1-A). All except two of these parasites were originally derived from cattle in Kenya, Uganda, Zimbabwe and Zambia: the two exceptions were from buffalo, one from Ol Pejeta ranch, Nanyuki, Kenya and the second from an animal experimentally infected with a parasite originating from a buffalo in Serengeti National Park Tanzania [21], [22]. Stabilate 4108 was prepared following tick passage from the Marikebuni *T. parva* stabilate 3014 [21]. Three cell lines (LS6, 7 and 8), BN64 Muguga, BN65 Kiambu 5 and BN140 Serengeti [23], [24] were generated by infecting lymphocytes with sporozoite seed stabilates used in the production of a large batch of the Muguga trivalent cocktail employed for vaccination by infection and treatment [25].

#### (ii) Cattle-derived (CD)

A further 27 cell lines (Table S1-B) were isolated directly from clinically reacting infected cattle in three regions of Kenya between 1997 and 2000. Kilifi in coast province; Kakuzi in central province [26] and Nyairo in the Trans-Nzoia district in western Kenya [15]. Two cell lines cloned by limiting dilution (CD4 and CD5) were derived from a mixed parasite isolate from Kakuzi [26].

#### (iii) Buffalo-derived (BD)

A third set of 16 isolates was obtained directly from buffalo (Table S1-C). Ten isolates (BD1 to 9 and BD13) were obtained by culture of leukocytes from blood samples collected from buffalo in the Masai Mara game reserve and Trans-Mara, Kenya [27], [28]. Isolate D10 was obtained from a buffalo infected by application of ticks from Ngong near Nairobi. Isolate BD11 was from buffalo 5641 born in captivity at the Central Veterinary Laboratories (CVL, Nairobi) and not exposed to *Theileria* prior to infection with *T. p. lawrencei* stabilate 177 [27]. BD12 and 14 represented parasites originating from Laikipia district in central Kenya while BD13 and 15 were from Mara, and all held at the CVL in Nairobi. For number BD16, PBMC from clean buffalo 7344 was infected with stabilate 3081 prepared from buffalo 7014, followed by cloning as described [21], [29].

#### (iv) Buffalo-associated (BA)

The fourth set of 22 isolates was obtained from cattle that grazed in close proximity to buffalo at Marula farm in Naivasha, Kenya (Table S1-D). The cattle were part of a trial performed in the year 2000 to investigate protection afforded to immunized cattle in an area where cattle and buffalo were co-grazing. The cattle were immunized by infection and treatment (ITM) with either the FAO1 Muguga cocktail stabilate, or one of two different Marikebuni stabilates 316 and 3014 (a parent stabilate of 316). Un-immunized control cattle were also exposed to *T. parva* challenge. Following vaccination, cattle were allowed to graze in close proximity to buffalo. They therefore received a *T. parva* challenge from ticks that may have recently fed on buffalo. In the study reported here, cell lines were obtained from lymph node biopsies from 22 of these cattle during the acute reaction phase, which exhibited the typical clinical pattern of low parasitosis and parasitaemia associated with infection by parasites originating from buffalo [1]. The majority of these parasites contained a 129 bp sequence insert within the p67 sporozoite antigen gene, assayed by PCR (Bishop R, Spooner P, Musoke AJ, Odongo D, unpublished), which is typical of *T. parva* parasites of buffalo origin in East Africa [30].

### CD8 epitopes present within the selected antigen genes

We have previously identified two antigens 543 and 174 amino acids long, Tp1 and Tp2 respectively, that are recognised by bovine CD8^+^ T cells (GenBank accession nos. XP_762973 and XP_765583). This was achieved by direct screening of a *T. parva* Muguga schizont cDNA library with CD8 T-cell lines. A single CD8^+^ T-cell epitope (VGYPKVKEEML: Tp1_214–224_) has been mapped in the Tp1 antigen [16]. Six distinct CD8 T-cell epitopes, SHEELKKLGML- Tp2_27–37_, DGFDRDALF- Tp2_40–48_, KSSHGMGKVGK- Tp2_49–59_, FAQSLVCVL- Tp2_96–104_, QSLVCVLMK- Tp2_98–106_ and KTSIPNPCKW- Tp2_138–147_, presented by 5 different class I alleles have been mapped in Tp2 [17], [31], [32].

### Antigen cloning and sequencing strategy

Specific forward and reverse primers were designed for PCR amplification of Tp1 and Tp2 gene sequences; one primer pair amplified a 432 bp region located between nucleotides 523 and 954 of the Tp1 ORF containing the known CD8 T-cell epitope (Tp1 forward primer: 5′-ATGGCCACTTCAATTGCATTTGCC-3′; Tp1 reverse primer: 5′-TTAAATGAAATATTTATGAGCTTC-3′) containing a tagged stop codon underlined; a second primer pair was used to amplify 525 bases comprising the complete Tp2 ORF (Tp2 forward primer: 5′-ATGAAATTGGCCGCCAGATTA-3′; Tp2 reverse primer: 5′-CTATGAAGTGCCGGAGGCTTC-3′). Total genomic DNA from *T. parva*-infected lymphocytes was prepared as recommended [33]. Genomic DNA (30 ηg) was PCR amplified in a 33 µl reaction with 25 U/ml AmpliTaq Gold DNA polymerase (Applied Biosystems, USA) in the presence of 33 ηg each of specific forward and reverse primers, 1× PCR Gold buffer (Applied Biosystems) containing 1.5 mM MgCl_2_ and 200 µM dNTP and using a programmable thermal cycler (MJ Research, Watertown, MA, USA). The cycling conditions were: step 1, 95°C for 11 min; step 2, 95°C for 30 s; step 3, 50°C for 45 s; step 4, 72°C for 30 s (30 times from steps 2 to 4); step 5, 72°C for 10 min. 7 µl of the PCR products was analyzed by electrophoresis in ethidium bromide-stained 1.5% agarose gels as described [33]. All the samples studied were positively amplified by PCR. For sequencing, 10 µl of the PCR products was treated with 10 U of exonuclease I and 1 U of shrimp alkaline phosphatase (United States Biochemical, USA) at 37°C/15 min, followed by 15 min incubation at 80°C. 4 µl of treated PCR product was sequenced directly or following cloning into pGEM-T Easy vector (Promega, USA) using specific primers and an ABI 3730 capillary sequencer (Applied Biosystems).

### Predicted amino acid sequences

Open reading frames present within the sequences generated from the amplified DNA fragments were translated into amino acid sequences using EMBOSS-Transeq software [34] and converted into FASTA format. Alignments of nucleotide and amino acid sequences were performed using CLUSTALW version 1.83 [35].

### Genetic diversity and population structure

The evolutionary genetic distances (expressed in terms of the number of differences per 100 bases or amino acids, including length polymorphisms) between every pair of sequences in a multiple alignment were generated using the DISTMAT program accessible at http://hpc.ilri.cgiar.org/emboss/ [36]. Estimates of DNA polymorphism, *π*, determined as the average number of nucleotide differences per site, were obtained with DnaSP v5 [37]. The Excel plug-in ‘Genalex6’ [38] was used to perform Principal Component Analysis (PCA) based on these distances. Analysis of molecular variance (AMOVA) was performed using ‘Genalex6’ [38] in order to investigate the distribution of genetic variation among allelic sequences and to determine the level of population differentiation. Pair-wise estimates of genetic distance among populations within the species were calculated using Φ_PT_, the proportion of variance among populations relative to total variance.

### Molecular evolution

The unrooted phylogenetic tree for each locus was estimated by neighbor-joining as implemented in MEGA [39]. The mode and intensity of selection, *ω* (∼*d*_N_/*d*_S_, the ratio of non-synonymous to synonymous substitution rates), acting on each locus was estimated using a codon-based substitution model [40] as implemented in PAML's program *codeml* [41]. Several models of evolution, namely M0, M1a, M2, M3, M7 and M8 [42] were tested to determine which provided a better fit to the data, given the phylogenetic relationships among sequences. Model M0 assumes a single rate of evolution for all codons (sites); all other models implemented allow for variable *ω* values across sites; however they do not allow *ω* to vary among branches of the phylogeny. For each model analyses were run twice, with different *ω* start values, to account for the possibility of sub-optimal peaks in the likelihood function. In each case, the analysis with the highest likelihood score is reported. A likelihood ratio test (LRT) was used to determine the significance of the difference in likelihood value of pairs of nested models, in which one allows for positive selection and the other does not (namely, M2 *vs.* M1a, and M8 *vs.* M7). In each case the test statistic (two times the difference in likelihood of two models of evolution x and y, 2Δ*lnL*_My-Mx_) was evaluated against a chi-square distribution with 2 degrees of freedom. All analyses were launched and monitored, and results visualized, using IDEA [43].

## Results

In order to determine the level of genetic diversity in isolates of *T. parva* obtained from cattle and buffalo in regions of Africa where ECF is endemic, sequence polymorphism analyses of two CD8 T cell target antigens, Tp1 and Tp2, were conducted. Identifiers prefixed with LS or CD denote cattle parasites with no association with buffalo, while BD or BA denote those from buffalo or cattle with association with buffalo (see Materials and Methods for details).

### The Tp1 locus

**Figure 1:**
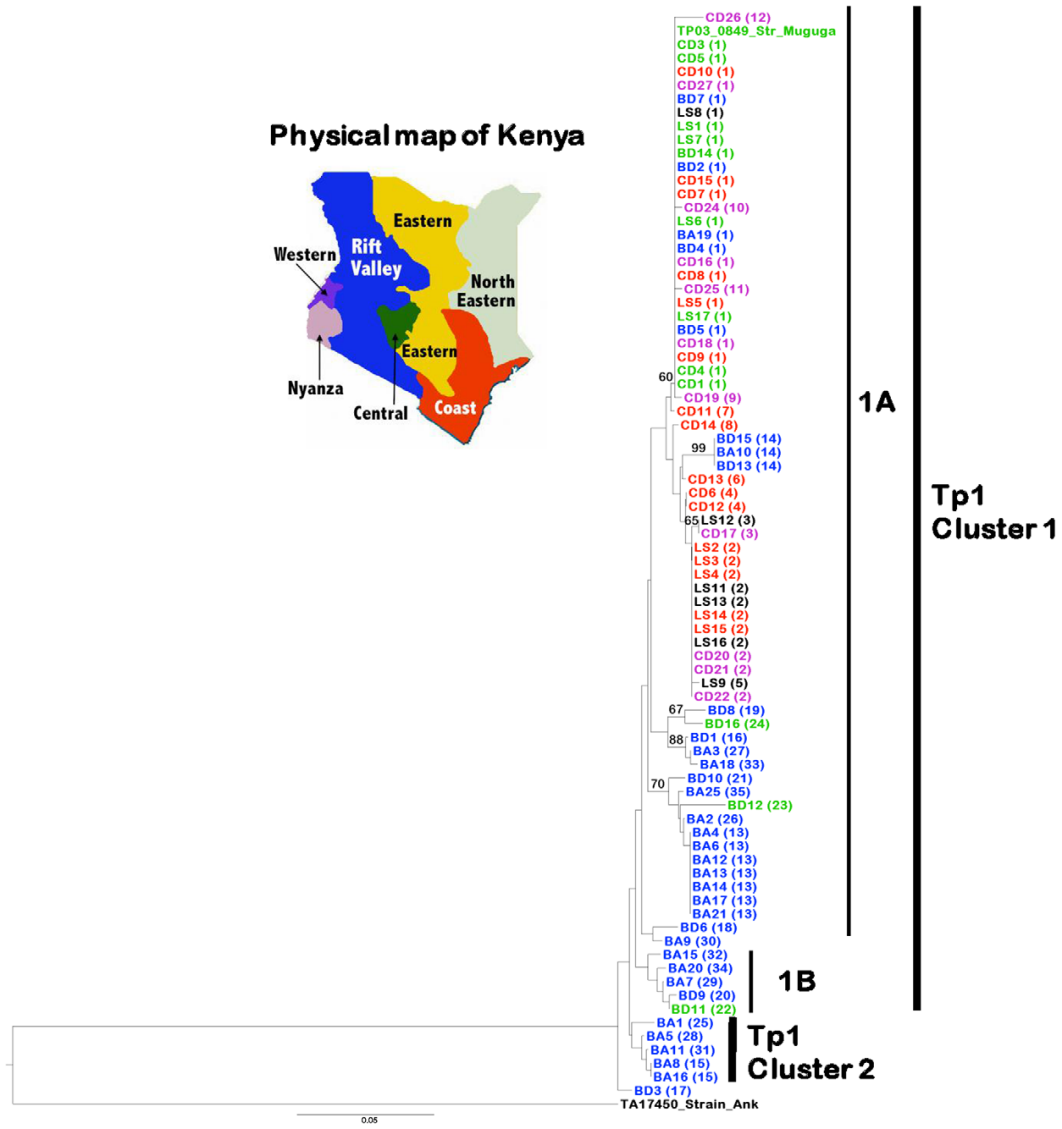
Neighbour-joining tree showing relationships among 79 cattle and buffalo-derived *T. parva* isolates. These data are based on the sequences of 35 Tp1 gene alleles obtained in this study. *T. annulata* (TA17450 Strain_Ank) was used as an outgroup to root the tree. Isolates with their corresponding alleles in parentheses are indicated and colour-coded based on their geographical location in Kenya. Isolates from outside Kenya (Tanzania, Uganda, Zambia and Zimbabwe) are depicted in black. The TP03_0849 gene from the *T. parva* (Muguga) genome sequence was also included in the analysis. Bootstrap values >50% are shown above branches. A Major clade and minor clade with two sub-clusters is indicated.

The 432 bp region of Tp1 that was sequenced is located in the centre of the gene and extends from nucleotides 523 to 954 of the reference Tp1 sequence (accession number XP_762973), corresponding to sample LS1 (Table S1). This region encodes 144 amino acids or 26.5% of the 543-residue Tp1 protein. We identified 35 different alleles of the Tp1 gene in the 79 parasites whose sequence was determined (Tables S1 and S2); overall polymorphism in the region was *π* = 0.019 (or 1.9%). We were unable to determine the Tp1 sequence from 3 of the 82 isolates. The alleles were distinguished by single nucleotide polymorphisms (SNPs) at 51 nucleotides, and two in-frame indels of 36 and 12 nucleotides, respectively (Figure S1). Compared to the reference sequence, deletions were present in 22 isolates, among which there were three buffalo-derived parasites (BD3, 8 and 16) in which both deletions were observed. Allele 1, which is present in the *T. parva* Muguga reference sequence (LS1), was represented in 24 of the 79 isolates (30.3%). The alleles that were genetically most distant from the Muguga sequence were alleles 17, 19 and 24, found in BD isolates. These shared a genetic distance of 24.82% from allele 1. However, this was heavily influenced by the presence of a deletion of 36 nucleotides in these isolates (Figure S1). The largest observed genetic distance of 26.76% was between BD isolates 10 and 16 that were derived from buffalo in south-western Kenya (Mara) and central Kenya (Laikipia), respectively (Fig. 1). In comparison, the greatest genetic distance observed between any pair of isolates derived from cattle that had no contact with buffalo was only 2.3% (isolates CD26 versus CD17, LS9 and LS12).

We observed that four different Tp1 alleles (1, 2, 3 and 5) were present among the 16 LS parasites (derived originally from nine stocks from five countries), 11 different alleles among the 25 CD stocks from Kenya, 11 among the 16 Kenyan BD stocks, and 15 among the 25 BA stocks that came from a single farm (Table S1; Fig. 1). A total of 12 different alleles was obtained from the 41 LS and CD parasites, and overall polymorphism in these isolates was *π* = 0.7%. In contrast, there were 24 different alleles from the 41 BA and BD stocks studied, with *π* = 2.2%. This suggests that there is greater diversity within the Tp1 locus among parasites in buffalo than in those maintained in cattle.

**Figure 2:**
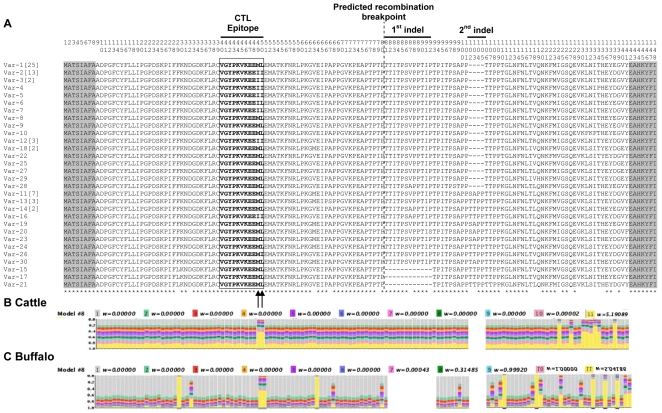
Multiple amino acid sequence alignment of the 30 Tp1 antigen variants obtained in this study. (**A**) The single letter amino acid code is used throughout. Variants named Var1-30. Residue coordinates are listed above the alignment. The single currently identified CD8 T-cell target epitope (coordinates 40–50 bolded and boxed) and the positions of the two indels are indicated. The two polymorphic residues in the T-cell epitope are indicated by arrows. A predicted recombination breakpoint between amino acid residues 80 and 81 is indicated with a broken vertical lane. The frequency of each variant is indicated in square brackets, when larger than 1. Residues conserved in all sequences are identified below the alignment (*). The flanked PCR primers regions not included in estimations of % residues conserved are shadowed. The distribution of selective constraints across the Tp1-encoded peptide, according to model M8, is shown for cattle (**B**) and buffalo (**C**) parasites. The stacked bar chart above each amino acid residue shows the probability that the degree of selective constraint on that residue falls into each of the several *ω* classes indicated by the color legend.

The predicted protein sequences of the Tp1 alleles were compared and the nucleotide variations described above resulted in 30 distinct protein variants (Table S2), due to amino acid changes at 27 residues, among which 4 were located in the indel regions (Fig. 2). The breadth of variation at the protein level reflected that observed for the alleles, in that greater diversity was present in the BA and BD isolates. Only one allele (variant 1 which was present in the Muguga reference sequence) was found in all four groups (Table S1).

Of particular interest was a comparison of the defined CD8^+^ T-cell epitope (VGYPKVKEEML) located within the sequenced region of Tp1. We observed three variants of the epitope resulting from substitutions in the two carboxy-terminal amino acids (Fig. 2). The three new variant epitopes ended in -MI, -IL or –II, as indicated in Table S1. The majority of isolates analysed (58 out of 79) displayed the epitope sequence (-ML) present in the *T. parva* Muguga reference isolate, including 36 of the 41 BA and BD isolates. The next most common variant (-II) was observed in 21 cell lines, and was strongly associated with isolates derived from cattle (LS and CD). The single substitution variants (-IL and –MI) were observed in two CD and one BA cell line, respectively.

### The Tp2 locus

**Figure 3:**
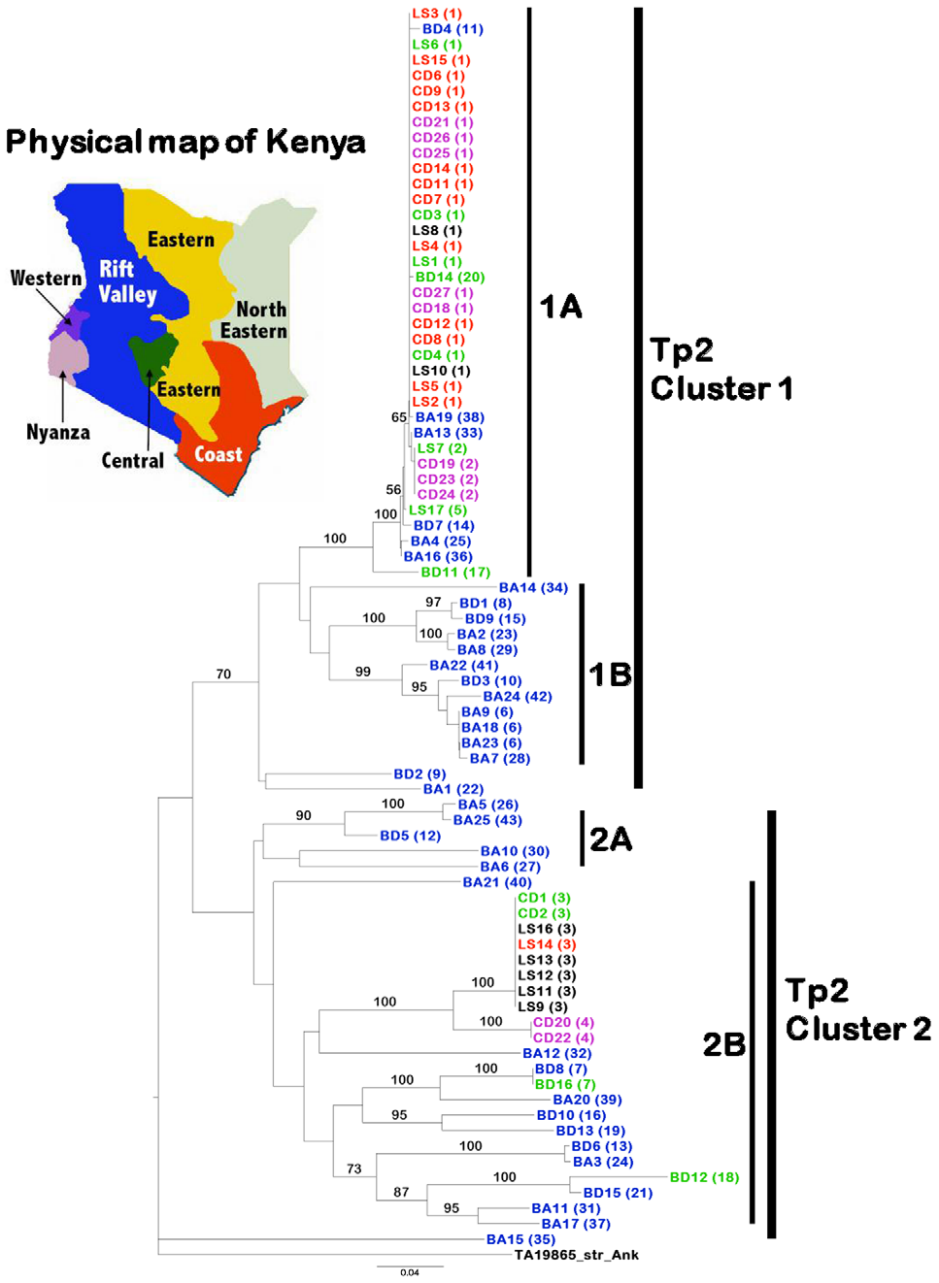
Neighbour-joining tree showing relationships among 80 cattle and buffalo-derived *T. parva* isolates. These data are based on the sequences of 43 Tp2 alleles obtained in this study. The Tp2 homologous from *T. annulata* (TA19865_Strain_Ank) was used as an outgroup to root the tree. Isolates with their corresponding alleles in parentheses are indicated and colour-coded based on their geographical location in Kenya. Isolates from outside Kenya (Tanzania, Uganda, Zambia and Zimbabwe) are shown in black Bootstrap values >50% are shown above branches. Two major clades and sub-clusters within these are indicated.

The full-length Tp2 gene sequence in the reference Muguga genome sequence (XP_765583), predicted to encode a protein of 174 amino acids, was sequenced in 77 isolates. We were unable to determine the sequence from five isolates. As four Tp2 sequences were identified in the Marula buffalo-associated isolate N106 (BA21 to 24), a total of 80 Tp2 sequences were analyzed (Table S1). We identified 43 alleles among the sequenced isolates, with SNPs observed at 320 nucleotides (Fig. 3 and Figure S2), and overall *π* = 17.9%, almost an order of magnitude higher than in the Tp1 segment analyzed. No indels were detected. Among the cattle-derived isolates, four different alleles were present among the 17 LS isolates. Three of these alleles, plus a fifth were observed among the 22 CD isolates. In contrast, 15 different alleles were detected among the 16 BD parasites and 23 different alleles were identified among the 25 BA isolates. Thus among 39 isolates from cattle that were not associated with buffalo only 5 Tp2 alleles were observed (with allele 1 from the Muguga reference sequence the most common, being present in 24 of the 39 isolates (61.5%)), while among 41 isolates or clones of likely buffalo origin there were 38 alleles (Table S1). The BA and BD parasites shared no alleles with the LS and CD stocks. The Muguga allele was not present in any of the BD or BA parasites, although the allele from BA19 differs by a single, synonymous nucleotide substitution. The sequence of BD12 was genetically the most distant from the Muguga reference sequence, the genetic distance being 29.71%. The largest genetic distance of 31.24% was observed between BA15 and two other buffalo related isolates, BA17 and BD13. In contrast, isolates BD8 and 16 had identical Tp2 alleles (Fig. 3; Table S1). This was also the case for BA9, 18 and 23 although they carried different Tp1 alleles. The greatest genetic distance between any pair of cattle-derived isolates was 24.95%, which is 10-fold greater than the maximum genetic distance observed between any pair of Tp1 sequences from cattle.

**Figure 4:**
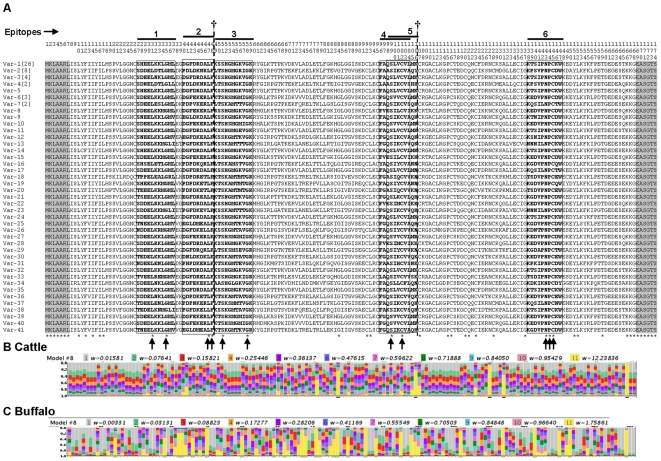
Multiple amino acid sequence alignment of the 41 full-length Tp2 antigen variants obtained in this study. (**A**) The single letter amino acid code is used throughout. Variants named Var1-41. Residue coordinates are listed above the alignment. The defined epitopes that are recognized by CD8 T cells (bolded and Boxed) are overlined and numbered from 1 to 6. The conserved amino acid residues in the epitopes are indicated by arrows. The frequency of each variant is indicated in square brackets, when larger than 1. Conserved residues are identified below the alignment (*). (†) denotes the two predicted recombination breakpoints, the first at the junction of epitope 2 and 3 (between residues 48 and 49) and the second at the C-terminal margin of epitope 5 (between residues 106 and 107). The flanked PCR primers regions not included in estimations of % residues conserved are shadowed. The distribution of selective constraints across the Tp2-encoded peptide, according to model M8, is shown for parasite isolates from cattle (**B**) and buffalo (**C**); legend as in Fig. 2.

When the predicted protein sequences were compared, we observed that the 43 alleles yielded 41 protein variants (Fig. 4). The cattle-derived LS and CD isolates contained only four protein variants, with the remaining 36 variants being present in the BA and BD isolates (Fig. 3; Table S1). As for Tp1, the results suggest that there is much greater diversity in the Tp2 sequences among the isolates obtained from buffalo and from cattle grazing with buffalo, than those derived from cattle. Whilst no alleles were found which were common to the two groups (LS and CD versus BA and BD), in some instances the predicted protein sequences were identical or very similar. For example, the Tp2 antigen from buffalo 7014 and Marula buffalo-associated isolate N102 (BD14 and BA19) was identical to that of the reference Muguga parasite, isolated from cattle. The Tp2 antigen from Mara buffalo 6998 (BD7) differed by one amino acid residue at the C-terminal end from the Muguga reference Tp2 protein (variant 1 vs variant 14, Fig. 4).

Six different CD8^+^ T-cell epitopes restricted by five different class I MHC haplotypes have been identified in the Tp2 protein (Fig. 4). The Tp2 sequences described here revealed many variants for each epitope, ranging from 18 for epitope number six to 25 for epitope number one (Table 1). In five epitopes, substitutions were observed at all but two of the amino acid residues. The exception was epitope 6 where NPC (residues 6–8) was present in every sequence (Fig. 4). The most diverse single residue was position 2 in epitope 6 (coordinate 139 in the protein alignment), where eight different amino acids were noted in the 18 variant sequences (Table 1). Except for epitope 1, the Tp2 epitopes of the *T. parva* Muguga isolate were also the most commonly observed variants among all the isolates analysed here. As indicated above, this was also the case for the Tp1 epitope.

**Table 1:** Tp2 CTL epitopes variants obtained in this study.

Epitope Variants	Epitope 1 (Tp2_27–37_) (25 variants)	Epitope 2 (Tp2_40–48_) (21 variants)	Epitope 3 (Tp2_49–59_) (23 variants)	Epitope 4 (Tp2_96–104_) (19 variants)	Epitope 5 (Tp2_98–106_) (19 variants)	Epitope 6 (Tp2_138–147_) (18 variants)
V1	SHEELKKLGML 1, 5,11,14	DGFDRDALF 1, 2, 5, 11, 14, 17, 22, 24, 28, 32, 35	KSSHGMGKVGK 1, 2, 5, 14, 17, 24, 28, 32, 35	FAQSLVCVL 1, 2, 5, 11, 14, 17, 24, 32, 35	QSLVCVLMK 1, 2, 5, 11, 14, 24, 32, 35	K**T**SIPNPCKW 1, 2, 5, 11, 14, 24, 32, 35, 38
V2	SDEELNKLGML 2, 34	PDLDKNRLF 3, 4	LTSHGMGRIGR 3	FAASIKCVA 3	ASIKCVAQY 3	K**P**SVPNPCDW 3, 4
V3	SDDELDTLGML 3	DGFEKERLF 6, 8, 10, 15, 27, 39, 40	LTSHGMGKIGR 4	LAASIKCVS 4	ASIKCVSHH 4	K**E**DVPNPCDW 6, 8, 10, 15, 22, 27, 28, 40
V4	SDNELDTLGLL 4	PDPVKERLF 7	KTSHSMGMIGK 6, 10, 27, 39, 40	FGQSVVCVL 6, 10, 27, 39, 40	QSVVCVLMR 6, 10, 27, 39, 40	K**Q**SILNPCTW 7, 17
V5	SDEELKKLGML 6, 10, 17, 24, 27, 32, 35, 39	EGLDKDALF 9	LTSKAMTTVGK 7	FAQSIKCVS 7, 37	QSIKCVSQH 7, 37	KTNIPNPCDW 9
V6	SDEELESLGML 7	EGFDKEALF 12	RSSHGMGKVGK 8, 15	FVESILCVI 8, 15	ESILCVIKK 8, 15	K**S**NIPNPCKW 12
V7	SDEELKKMGML 8, 15	PGFDKEVLF 13, 23	KSSKGMGKVGK 9	FAQSIKCVA 9	QSIKCVAQH 9, 25, 41	N**N**NILNPCKW 13, 23
V8	SNEELKKLGMV 9, 12	PDPDKQRLF 16	KSSHGMGEVGK 11	FGQSIKCVA 12, 25, 41	QSIKCVAQK 12	K**G**DVPNPCQW 16, 19
V9	SDDELKKMGLI 13, 23	SDPDRETLF 18	KSSKGMTKVGK 12	FGQSIKCVV 13, 23, 30, 36	QSIKCVVQK 13, 23, 30, 36	TSDVPNPCEW 18, 20
V10	SHSELETLGML 16	PDPDKETLF 19, 20	KSSKAMTTTGK 13, 23	YAQSIYCVA 16	QSIYCVANN 16, 19	KGDVPNPCDW 21, 25, 41
V11	TPPELEALGRL 18	EGFDREALF 21	LTSKAMSTVGK 16	FGASIQCVV 18, 20	QSLVCVLMN 17	K**D**NTPNPCNW 26
V12	SHEELKKMGML 19	EGLDMEALF 25, 41	KTSKAMTMTGR 18, 19, 20	CFAQSIYCVA 19	ASIQCVVKN 18, 20	KGDAPNPCTW 29
V13	TEEELKRMGML 20	DGFDKELLF 26	KTSNGMTKVGK 21	FGQSLKCVL 21	QSLKCVLQH 21	KEDVPNPCEW 30, 36
V14	TSEELKKLGMV 21	DGFDRQRLF 29	KSSHGMGKVGR 22	FVESIMCVI 22, 28	ESIMCVIKK 22, 28	KPKIPNPCDW 31
V15	SDDELRKLGML 22, 28	DNLDKDKLF 30	KTSKGMTKVGR 25, 41	FAQSIYCVV 26, 29	QSIYCVVKN 26, 29	TSDIPNPCKW 33
V16	TNEELKKLGMV 25, 41	PHPDKERLF 31	KSSKGMTAVGK 26, 38	FAASIHCVS 31	ASIHCVSNK 31	KDKIKNPCDY 34
V17	NDDELKRMGMV 26	DGFDRELLF 33	LTSKSMSEVGR 29	LAQSIVCVV 33	QSIVCVVSK 33	KQSILNPCNW37
V18	SDEELKELGML 29	PDFEKEKLF 34	MTSKAMTATGR 30	FAQSLLCFL 34	QSLLCFLQN 34	KEDVPNPCKW 39
V19	TEDELKKLGMV 30	QDPDKETLF 36	KTSKGMTEVGK 31	FAKSIKCVS 38	KSIKCVSQH 38	
V20	NDEELENLGML 31	PDPNKERLF 37	KSSHGMGKIGR 33			
V21	TVEELREMGMV 33	PGFDKELLF 38	AASHGLGKVGK 34			
V22	SDDELNKLGML 36		KTSKAMTATGR 36			
V23	TDEELENLGML 37		LTSKSMMTVGK 37			
V24	TEDELKNMGLI 38					
V25	SDEDLKKLGML 40					

Note: Numbers following the epitope sequences are the corresponding antigen alleles (see Table S1) carrying the epitopes. Up to 8 different amino acid residues (in bold and underlined) are used in the second amino acid position in epitope 6.

Comparison of the two antigens revealed more alleles for the Tp2 gene than for Tp1 (Table S1). This was mostly due to the greater diversity of Tp2 genes found in the BA and BD isolates in which there were 38 different alleles out of a total of 43 sequenced, as compared to 5 in the 39 LS and CD isolates. By contrast in the cattle-derived isolates, there were 12 different Tp1 alleles out of a total of 35 alleles present in the complete dataset of all isolates. Overall, alignment of sequences from 80 isolates showed that only 45 of the 174 amino acid residues (25.8%) of Tp2 were conserved among alleles (Fig. 4). In contrast, 81.7% of amino acid residues are conserved among Tp1 alleles (Fig. 2).

### Molecular evolution analysis of the Tp1 and Tp2 genes

In order to characterize the evolutionary forces acting on these two loci, we estimated *ω* (∼*d*_N_/*d*_S_), the ratio of non-synonymous to synonymous substitution rates, which provides a measure of the mode and intensity of selection. For each locus the isolates were divided into two groups, one containing parasites obtained from cattle (CD and LS), and the other all parasites derived from, or associated with, buffalo (BD and BA, respectively). Six models of evolution (M0, M1a, M2, M3, M7 and M8) were tested (see Methods), to determine which among them provides a better fit to the patterns of variation observed across each gene. While M0 is clearly an oversimplification for most proteins, it provides a good benchmark against which more complex models can be compared. Three of the models tested (M2, M3 and M8) allow a subset of residues to evolve under positive selection. The comparison of two of those models, M2 and M8, with models that assume the absence of positive selection (M1a and M7, respectively) using a likelihood ratio test (LRT) provides a direct assessment of the likelihood of positive selection (see Methods).

#### (i) The Tp1 gene

The evolution of the Tp1 locus from both cattle and buffalo parasites is best described by models that allow positively selected residues, namely M2, M3 and M8, as indicated by higher log likelihood values relative to the other models (Table 2). Model M3 has the highest likelihood score for both cattle and buffalo parasites. This model allows one subset of residues to evolve under positive selection with *ω* estimated from the data (*ω*_2_), and the remaining residues grouped into two classes 0 and 1, each with *ω*≤1 estimated from the data (*ω*_0_ *and ω*_1_). In the case of cattle parasites, 80% of residues fall into classes 0 (*p*_0_ = 0.77) or 1 (*p*_1_ = 0.03), both of which evolve under extreme purifying selection (*ω*_0_≈*ω*_1_≈0) (Table 2). The remaining 20% of residues (*p*_2_ = 0.20) appear to evolve under strong positive selection, with non-synonymous polymorphism occurring about five times more frequently than synonymous polymorphisms (*ω*_2_∼5.2). Ten of these residue positions show statistically significant evidence for positive selection, and two of them (sites with coordinates 49 and 50 in the protein alignment) fall within the known Tp1 epitope (Table 2, Fig. 1b). Models M2 and M8 have nearly identical likelihoods to model M3 (all have lnL∼−617.5). Model M2, similarly to model M3, groups residues into three classes, but one of those classes, class 1, is composed of residues assumed to be strictly conserved (*ω* = 0). Model M8 is an approximation to a continuous distribution, with residues partitioned into 10 classes, each with *ω*<1, and another class with *ω*≥1. Averaged across the whole segment of the gene, and according to the three most likely models (M2, M3 and M8) the number of synonymous and non-synonymous polymorphisms per site is roughly identical (*ω* = 1.07), suggesting either the absence of selective constraints (*ω* = 1) or positive selection (*ω*>1). To distinguish between these scenarios we used a likelihood ratio test of nested models. When M2 is compared to M1a, and M8 to M7, the models M2 and M8, which allow positive selection, do not provide a significantly better fit to the data than M1a and M7 (2Δ*lnL*_M2-M1a_ = 3.11, *P*∼0.21; 2Δ*lnL*_M8-M7_ = 3.15, *P*∼0.21), suggesting that the accumulation of non-synonymous mutations may be due to the absence of selective constraints. As discussed above, the cattle sequences are very similar to each other; in fact, they are clustered in only two clades, each with a very recent common ancestor (Fig. 1).

**Table 2:** Evolution of the Tp1 locus: likelihood value (lnL) and parameter estimates for six evolution models implemented.

Model^a^	lnL	*d*_N_/*d*_S_ b	Estimated parameter values^c^
**CD+LS isolates**			
M0 (one-ratio)	−620.2986	0.91	*ω* = 0.91
M1a (nearly neutral)	−619.0294	0.55	*p*_0_ = 0.45, (*p*_1_ = 0.55), *ω*_0_ = 0.00
M2 (positive sel.)	−617.4723	1.07	*p*_0_ = 0.79, *p*_1_ = 0.0, (*p*_2_ = 0.21)
			*ω*_0_ = 0.00, *ω*_2_ = 5.19
			Positively selected sites (BEB): 124, 133, 138
M3 *(discrete)*	*−617.4722*	1.07	*p*_0_ = 0.77, *p*_1_ = 0.03, (*p*_2_ = 0.20)
			*ω*_0_ = 0.00, *ω*_1_ = 0.00, *ω*_2_ = 5.19
			Positively selected codons (NEB): 49**, 50**, 124**, 127**, 130**, 131**, 132**, 133**, 134**, 138**
M7 (beta)	−619.0489	0.50	*p* = 0.005, *q* = 0.005
M8 (beta & *ω*)	−617.4725	1.07	*p*_0_ = 0.79, (*p*_1_ = 0.21), *p* = 0.005, *q* = 1.702
			*ω* = 5.19
			Positively selected sites (BEB): 49, 50, 124, 127, 130, 131, 132, 133, 134, 138
**BA+BD isolates**			
M0 (one-ratio)	−892.7489	0.49	*ω* = 0.49
M1a (nearly neutral)	−876.3387	0.29	*p*_0_ = 0.71, (*p*_1_ = 0.29), *ω*_0_ = 0.00
M2 (positive sel.)	−870.9605	0.59	*p*_0_ = 0.72, *p*_1_ = 0.21, (*p*_2_ = 0.07)
			*ω*_0_ = 0.016, (*ω*_1_ = 1.0), *ω*_2_ = 5.13
			Positively selected sites (BEB): 29, 63, 110, 118**, 132, 135, 138
*M3 (discrete)*	*−870.9524*	0.58	*p*_0_ = 0.68, *p*_1_ = 0.25, (*p*_2_ = 0.07)
			*ω*_0_ = 0.00, *ω*_1_ = 0.84, *ω*_2_ = 4.98
			Positively selected codons (NEB): 29, 63, 110, 118**, 132, 135, 138
M7 (beta)	−876.3474	0.30	*p* = 0.005, *q* = 0.012
M8 (beta & *ω*)	−870.9584	0.58	*p*_0_ = 0.93, (*p*_1_ = 0.07), *p* = 0.021, *q* = 0.066
			*ω* = 5.04
			Positively selected sites (BEB): 29*, 49, 50, 63*, 110*, 118**, 132, 135, 138

a Sites models of evolution. Nomenclature as in Yang *et al.* (2000). Model with highest likelihood for each locus is shown in italics.

b Weighted average of *d*_N_/*d*_S_ across all sites.

c Parameters in parenthesis are inferred (probabilities add up to 1); all others are estimated from the data. Significance levels indicate probability that *ω*>1 for the site:

*p≥0.95;

**p≥0.99 (no asterisks signifies *p*<95%). Significance levels for M3 is based on a Naïve Empirical Bayes (NEB) analysis, while that of models M2 and M8 is based in a Bayes Empirical Bayes (BEB) analysis (Yang, Wong, and Nielsen 2005).

In Tp1 sequences from buffalo parasites, and again according to model M3, 68% of the sites (*p*_0_) evolve under very strong purifying selection (*ω* = 0), while ∼25% of the sites have almost identical numbers of synonymous and non-synonymous polymorphisms (*ω*_1_ = 0.84). Finally, about 7% of the sites evolve under strong purifying selection, with *ω*_2_∼5. Seven of those sites show statistically significant positive selection, but none of them falls within the epitope region. Across the whole segment analyzed, according to model M3, *ω*∼0.6. Again, models M2 and M8 have nearly identical values of log likelihood (lnL∼−870.0) and *d*_N_/*d*_S_ (*ω* = 0.6) to model M3. However, in contrast to cattle parasites, in the case of the sequences determined from buffalo parasites the models allowing positive selection (M2 and M8) seem to provide a significantly better fit to the data than their nested models (M1and M7, respectively), with 2Δ*lnL*_M2-M1a_ = 10.76 (*P*∼0.046) and 2Δ*lnL*_M8-M7_ = 10.78 (*P*∼0.0046). This result suggests that positive selection has been a significant force in the evolution of this locus in buffalo parasites.

Overall, the results for Tp1 reveal a remarkably high proportion of non-synonymous polymorphisms, with *d*_N_/*d*_S_>0.5 in cattle and buffalo parasites, respectively. Interestingly, however, those polymorphisms are concentrated in a minority of sites, with strong purifying selection prevalent in 70% to 80% of the residues in buffalo and cattle parasites, respectively (Table 2, Fig. 1bB and 1C).

#### (ii) The Tp2 gene

Similarly to the observations for Tp1, the results for locus Tp2 from both cattle and buffalo parasites show that models that allow positively selected sites (namely, M2, M3 and M8) provide a better fit to the data than do models M0, M1a and M7, as implied by their log likelihood values (Table 3). Model M3 provides the best fit to the cattle parasite data, but M2 and M8 have just slightly lower log likelihood values. According to model M3, approximately 24% of the sites (*p*_1_+*p*_2_) evolve under positive selection, but for none of those sites is the probability of *ω*>1 larger than 95% (Table 3). Model M8 provides the best description for the evolution of buffalo parasite sequences, but with a likelihood value clearly higher than all other five models, including M2 and M3 (Table 3). According to this model, ∼12% of the sites fall within the class of positively selected residues, and the result is statistically significant for residue positions 46, 77, 91, 130 and 139 (Table 3, Fig. 4C).

**Table 3:** Evolution of the Tp2 locus: likelihood values (lnL) and parameter estimates for the evolutionary models implemented.

Model^a^	lnL	*d*_N_/*d*_S_^b^	Estimated parameter values^c^
**CD+LS isolates**			
M0 (one-ratio)	−1213.7784	0.45	*ω* = 0.45
M1a (nearly neutral)	−1205.9230	0.45	*p*_0_ = 0.66, (*p*_1_ = 0.34), *ω*_0_ = 0.17
M2 (positive sel.)	−1201.4097	0.86	*p*_0_ = 0.65, *p*_1_ = 0.33, (*p*_2_ = 0.02)
			*ω*_0_ = 0.20, *ω*_2_ = 19.41
			Positively selected sites (BEB): 83, 105, 126, 130, 165*
M3 *(discrete)*	*−1201.3067*	1.29	*p*_0_ = 0.76, *p*_1_ = 0.22, (*p*_2_ = 0.02)
			*ω*_0_ = 0.25, *ω*_1_ = 1.41; *ω*_2_ = 50.26
			Positively selected sites (NEB): 29, 34, 39, 40, 46, 49, 66, 69, 74, 77, 81, 83, 86, 91, 98, 101, 104, 105, 106, 122, 126, 130, 165
M7 (beta)	−1206.8250	0.47	*p* = 0.4024, *q* = 0.4527
M8 (beta & *ω*)	−1201.5758	0.78	*p*_0_ = 0.97, (*p*_1_ = 0.03), *p* = 0.695, *q* = 0.865
			*ω* = 12.24
			Positively selected sites (BEB): 83, 105, 126, 130, 164**
**BA+BD isolates**			
M0 (one-ratio)	−6194.3698	0.50	*ω* = 0.50
M1a (nearly neutral)	−5989.1350	0.54	*p*_0_ = 0.54, (*p*_1_ = 0.46), *ω*_0_ = 0.14
M2 (positive sel.)	−5974.5287	0.68	*p*_0_ = 0.50, *p*_1_ = 0.41, (*p*_2_ = 0.09)
			*ω*_0_ = 0.14, (*ω*_1_ = 1.0), *ω*_2_ = 2.24
			Positively selected sites (BEB): 46, 73, 77, 83, 91*, 118, 130*, 139**, 146, 149, 165
*M3 (discrete)*	*−5967.0899*	0.57	*p*_0_ = 0.41, *p*_1_ = 0.40, (*p*_2_ = 0.19)
			*ω*_0_ = 0.09, *ω*_1_ = 0.60, *ω*_2_ = 1.55
			Positively selected sites (NEB): 20, 34, 38*, 39, 40, 42, 46**, 56, 65, 68, 72, 73**, 74, 77**, 78, 82, 83*, 86, 91**, 94, 101*, 104, 105, 118*, 130**, 139**, 146**, 148, 149**, 165**
M7 (beta)	−5976.5388	0.46	*p* = 0.4114, *q* = 0.4877
M8 (beta & *ω*)	−5962.3507	0.57	*p*_0_ = 0.88, (*p*_1_ = 0.12), *p* = 0.487, *q* = 0.711
			*ω* = 1.76
			Positively selected sites (BEB): 20, 34, 38, 39, 42, 46*, 56, 65, 69, 73, 74, 77*, 78, 82, 83, 86, 91*, 94, 101, 104, 118, 130*, 139**, 146, 149, 165

**Note:** Legend as in Table 2.

Comparisons of the two nested pairs of models, M2 vs M1a and M8 vs. M7, show statistically strong evidence of positive selection in parasite sequences from cattle (2Δ*lnL*_M2-M1a_ = 9.03, *P*∼0.011; 2Δ*lnL*_M8-M7_ = 10.50, *P*∼0.005) and from buffalo (2Δ*lnL*_M2-M1a_ = 29.21, *P*∼4.5×10^−7^; 2Δ*lnL*_M8-M7_ = 28.38, *P*∼6.9×10^−7^). Unlike for Tp1 locus, the comparison between nested models is significant in cattle, Finally, the average *ω* is high in both cattle (0.78<*ω*<1.29) and buffalo (0.57<*ω*<0.68), as determined by the three most likely evolution models (Table 3).

In sharp contrast to what is observed for Tp1 (Fig. 2B–C), in which >50% of amino acid residues are extremely conserved (*ω* = 0), the Tp2 locus does not have a class of residues with an estimated *ω* = 0 (Fig. 4B–C) in either buffalo or cattle parasite sequences. Even though the average *ω* is about the same in both loci, polymorphism is an order of magnitude higher in Tp2 (*π* = 17.9%, compared with *π* = 1.9% in Tp1), and the observed variation widely distributed across the Tp2 locus, with the majority of sites either evolving relatively rapidly (*ω*>0.5) or under positive selection (*ω*>1). In spite of this difference, for both loci the average *ω* is larger than 0.5. Together with the evidence for positive selection in several residues, and the known antigenic role of the proteins encoded by these loci, these results suggest that positive selection has contributed significantly for the retention of non-synonymous polymorphisms in the parasite populations. Finally, it is of interest to note that, in both loci, the average *ω* value is slightly higher among cattle than in buffalo parasites.

### Distribution of *T. parva* antigen variability among parasite populations

We generated neighbour-joining trees for both loci rooted with the orthologous sequences from *T. annulata*, to examine whether the sequence diversity observed in Tp1 and Tp2 was associated with geographical origin or mammalian host species. In the case of Tp1, the phylogenetic analysis separated the 35 distinct alleles into a small clade (Tp1 cluster 2) containing 5 sequences originating exclusively from buffalo-associated parasites and a major clade (Tp1 cluster 1) containing two sub-clades 1A and 1B (Fig. 1). An exception was isolate BD3 which represented the single unique allele 17 that did not group with any of these clades or sub-clades. Allele 1 found in the reference Muguga F100 isolate and 67 other isolates, among which were the three component stocks of the FAO1 Muguga cocktail live vaccine (LS6, 7 and 8), was grouped with 23 other alleles into the subcluster 1A. This group contained 68 out of 79 isolates (86%) of both cattle and buffalo origins and from different geographical regions of Kenya and as well as from Tanzania (LS8), Uganda (LS12, 16), Zambia (LS9, 10) and Zimbabwe (LS11, 13).

**Figure 5:**
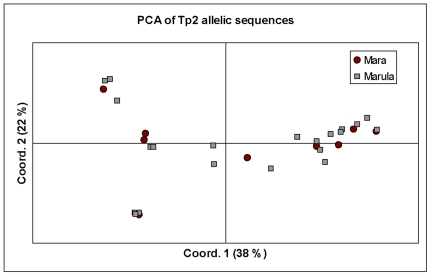
Principal component analysis of Tp2 allelic diversity. This diagram illustrates the relationship between buffalo-derived (Mara) and buffalo-associated, cattle-derived isolates (Marula), suggesting the isolates may belong to a single population. The proportion of variation in the dataset explained by the 1^st^ and 2^nd^ principal components is indicated in parenthesis.

The phylogenetic analysis of the 43 Tp2 distinct alleles also separated the 80 isolates studied into two major clusters (Fig. 3). Despite relatively large overall polymorphism among cattle isolates (*π* = 10.7%) most differences are attributed to variations between sequences in cluster 1 and cluster 2 (Fig. 5). Within each cluster the cattle parasites are nearly identical, and hence only 5 different alleles were found. In contrast, overall polymorphism among buffalo parasites was *π* = 21.2%. Most of the alleles were within cluster 1, which grouped into two subclusters 1A (37 isolates from LS, CD, BD and BA groups) and 1B (12 isolates, all derived from buffalo). The three isolates BD2, BA1 and BA15 encoding single unique alleles 9, 22 and 35 respectively, did not fall into any clade. Allele 1 found in the reference Muguga F100 isolate LS1 as well as in 23 other isolates was grouped with ten other alleles into the subcluster 1A. As was observed with the Tp1 subcluster 1A, the Tp2 alleles in cluster 1A were represented in parasites from different ecological regions throughout Kenya, including the coast (Mariakani, Marikebuni and Kilifi, with the isolates LS2-5; CD6-9, 11-14), the central region (Muguga, Kakuzi, Kiambu, Laikipia and Nanyuki, with the isolates LS1, 6, 7 and 17; CD3 and 4; BD11 and 14) and the western region/Rift valley (Nyairo and Masai Mara with the isolates CD18, 25-27, BD4). In addition, Tp2 alleles from an isolate from Zambia (LS10) and from the three component stocks of the FAO1 Muguga cocktail live vaccine (containing isolates from Kenya and Tanzania) were grouped into the 1A subcluster. Cluster 2 alleles comprised two subclusters, 2A (5 isolates, all derived from buffalo) and 2B (23 isolates from the LS, CD, BD and BA groups). The alleles in subcluster 2B were from parasites isolated from Kenya, Uganda, Zambia and Zimbabwe. The presence, for both loci, of large clades of nearly identical sequences from cattle isolates suggests a rapid spread among cattle.

We further analysed the partition of genetic diversity in the Tp2 locus, using AMOVA, by comparing the alleles from the Mara buffalo-derived (n = 10) parasites (BD) with those from the parasites isolated from buffalo-associated cattle (BA) (n = 25). All of the variation among Tp2 alleles in the locus was found to exist within each population, with none of the variation in the dataset attributable to differences between the populations (Φ_PT_ = 0). Thus, buffalo-derived isolates from Mara showed no evidence of differentiation from isolates from cattle at Marula farm that were presumed to have been infected by ticks that had fed on buffalo. The relationship between buffalo-derived (BD) and buffalo associated (BA) isolates is depicted using a PCA plot (Fig. 5).

Three remarkable observations result from these analyses. First, the laboratory (LS) and cattle-derived (CD) isolates represent a very small proportion of the variation in each locus, even though the samples were collected from across Kenya and surrounding countries. Secondly, for both Tp1 and Tp2, LS and CD isolates are grouped into two clades nested within more ancient lineages comprising the buffalo-derived and buffalo-associated parasites (Fig. 1 and 4c). Even though all buffalo-associated parasites (BA) were collected on a single farm within a short period of time, they displayed extensive sequence diversity in Tp1 and Tp2, suggesting that the vast majority of antigen variability resides in parasites maintained in buffalo. These data are consistent with the hypothesis that *T. parva* maintained in cattle represents a subset of the overall *T. parva* population present in buffalo and that buffalo act as a reservoir of antigenically diverse parasites that can be transmitted to cattle sharing grazing grounds with buffalo. Finally, despite the fact that the parasites examined were derived from two different mammalian hosts and from geographically distant sites, predominantly in Kenya but with a few isolates from other countries, the phylogenetic analyses suggest that they represent a single parasite population. More detailed population genetic studies using neutral loci are required to confirm this hypothesis.

## Discussion

The study revealed extensive allelic diversity in the Tp1 and Tp2 genes resulting in polymorphisms in all seven CD8 T-cell epitopes identified within these antigens. Overall polymorphism was one order of magnitude higher in Tp2 than in Tp1, and at least twice as high in buffalo than in cattle parasites. As expected, at the protein level the Tp2 antigen showed more extensive sequence diversity than Tp1 and both antigens displayed greater diversity in parasite isolates from buffalo or cattle grazing with buffalo compared to parasites from cattle in buffalo-free areas. While analyses of the sequences demonstrated evidence of selection for amino acid changes including residues within T cell epitopes, there was no significant enrichment for positively selected residues in the epitope sequences.

Nucleotide sequence analysis of part of the Tp1-encoding gene revealed 35 distinct alleles defined by point mutations and two regions containing indels. The predicted protein sequences identified 30 different variants of the antigen. Only one of these (variant 1, which is found in the reference *T. parva* Muguga isolate) was shared by the cattle-derived and buffalo-derived isolates. The single CD8^+^ T-cell epitope identified in Tp1 was relatively conserved at the amino acid level when compared to the six known Tp2 epitopes, with only four variants observed among 79 isolates, involving coding changes at residues 10 and 11 of the epitope.

Sequence comparisons indicated that the Tp2 antigen is highly polymorphic, with 43 distinct alleles and 41 predicted protein variants observed among the 80 Tp2 sequences obtained. The sequence variation was distributed across the gene and no deletions or insertions were observed. Analysis of the sequences encoding the Tp2 CD8^+^ T-cell epitopes revealed a very high number of amino acid substitutions, with only two or three amino acid residues being conserved in each of the six epitopes across the 80 *T. parva* isolates/clones studied. Eight different amino acids were found at a single residue position of one epitope (Table 1).

As might be expected from the difference in epitope number between the two loci, the frequency of variable residues was much higher in Tp2 than in Tp1. However, although there was evidence that positively selected codons were present in the T-cell epitopes, the epitope regions were not enriched for such codons compared to the rest of the antigen. Given that these antigens enter the class I MHC processing pathway and that cattle representing a relatively limited percentage of bovine haplotype space have so far been evaluated for the immunodominant *T. parva* epitopes that they present, it is likely that additional epitopes, recognised in the context of different MHC backgrounds are yet to be discovered. If the antigens contain a large number of additional epitopes, the analyses as conducted herein might fail to reveal significant enrichment of selection within the epitope sequences. Alternatively, these antigens may not be predominant targets of CD8^+^ T-cell responses in the buffalo, in which much of parasite evolution is likely to have occurred. A third explanation is that the host CD8^+^ T- cell response is not the major factor responsible for selection of diversification in these two proteins in *T. parva*. The Tp1 and Tp2 proteins have no identifiable orthologues in parasites other than *Theileria*, or any other taxa. Their functions remain unknown. Hence, these genes may be subject to alternative selective pressures, acting either on the schizont or other life cycle stages, such as those present in the tick vector, which result in the observed sequence diversity. If the above hypothesis were correct, escape of parasites from T cell recognition, in the mammalian host, would therefore be fortuitous, as has been proposed for both *Plasmodium* [44] and *Theileria* [45].

The sequence diversity data obtained in this study has provided further insight into the population structure of *T. parva*. The Tp2 alleles in parasites isolated from cattle that shared grazing with buffalo at a single farm in the Rift Valley of Kenya showed much greater sequence diversity than those from cattle where buffalo were not present, although these were derived from geographically distant sites. They were also indistinguishable from the alleles in the sample of buffalo-derived parasites. This strongly supports the assertion that parasites isolated from cattle grazing with buffalo originated from the buffalo reservoir. The observation that the diversity of Tp2 is considerably higher in buffalo (*π* = 21%) than in cattle not in contact with buffalo (*π* = 11%) suggests that variation in Tp2 evolved and is maintained primarily in the wildlife reservoir. The observed diversity may represent polymorphisms that accumulated in the wildlife (buffalo/tick) reservoirs over several millennia. This would have occurred well before the introduction of cattle into the region, estimated from archaeological records to be approximately six thousand years ago [46]. The limited diversity in the cattle population can be explained by a founder effect, in which only a subset of the buffalo parasite gene pool can be established and transmitted within the cattle population. Although the parasites isolated from cattle associated with buffalo are highly diverse, as illustrated by the level of polymorphism in the Tp2 gene in BA isolates, these parasites were isolated from lymph node biopsies obtained from clinically reacting animals. The cattle died with a typical buffalo-derived *T. parva* clinical syndrome involving low schizont parasitosis and a low piroplasm parasitaemia. This clinical picture (combined with the presence of insertions in the p67 sporozoite antigen gene characteristic of buffalo parasites [30]) suggests that the Marula BA parasites represent a sample of cattle-infective parasites present in the local buffalo population, rather than parasites that can sustainably be transmitted within the cattle population by ticks. Although it is not possible to make conclusive statements regarding the slightly higher average *ω* in cattle vs. buffalo parasites given the current sample size, it is interesting to speculate that it might be the result of more recently imposed selection associated with adaptation to a new host in evolutionary terms, relative to the buffalo. Alternatively, this difference may reflect the rapid expansion of the *T. parva* population among cattle, with a concomitant relaxation of purifying selection.

There is a striking contrast in the number of Tp1 and Tp2 variants observed among parasites isolated from cattle relative to buffalo. Of the 30 amino acid sequence variants identified in the partial sequence of Tp1, 10 are present in cattle not associated with buffalo, and nine of these are cattle-specific. By contrast only four out of 41 antigenic variants in Tp2 are present in cattle isolates and one of these was obtained only from a cattle maintained parasite (LS17) that was originally isolated from a buffalo. There are several possible explanations for this observation, including that subsequent to transfer from buffalo there has been (1) a relaxation of purifying selection in Tp1, (2) an increase in diversifying selection in Tp1 in cattle populations, or (3) an increase in purifying selection in the Tp2 locus. Finally, it is also possible that the founder population exhibited more diversity in Tp1 than Tp2, (4) either by chance or (5) because the allelic variants compatible with maintenance in cattle are more limited in Tp2 than in Tp1. The latter hypothesis (5), as well as (3), might imply that adaptation to cattle is influenced by the Tp2 allele sequence. The accumulation of both synonymous and non-synonymous mutations in cattle parasites sampled may reflect mostly an episode of relaxed selection associated with a rapid expansion of these isolates in the cattle population. Alternatively, the recent common ancestry of all cattle isolates may have resulted in lack of analytical power, due to insufficient time for new variants to arise and for the effects of small selection coefficients associated with slightly deleterious or advantageous mutations to be felt.

An interesting aspect of both Tp1 and Tp2 diversity is that the data suggest an epidemic population structure in cattle that are not co-grazing with buffalo, where a limited number of genotypes appears to have expanded and become over-represented in the population. For both loci, sequences from cattle parasites are clustered in only two clades, each composed of closely related l alleles. This population model has been observed previously in regional populations in Uganda, based on data from a panel of variable number tandem repeat (VNTR) polymorphic markers [14]. A more recent study demonstrated significant linkage disequilibrium within three regional populations in Kenya [15]. In both studies there was evidence of extensive genetic diversity among isolates but in Kenya there was no direct evidence of clonal expansion of particular genotypes. However, the analyses of the Kenyan populations were based on data from a large panel of VNTR polymorphic markers and did not include antigen gene sequencing; therefore the results are not directly comparable to those presented herein.

Allelic polymorphism of target antigens in natural populations of *T. parva* is a key issue for understanding the basis of strain specificity in immunity to the parasite. This study has focused on comparative analyses of the sequences of two genes that encode dominant CD8^+^ T-cell target antigens in field isolates of *T. parva* from cattle and buffalo. The principal aims were to provide information on antigenic diversity at the population level and to investigate whether the antigens are subject to positive selection for amino acid changes. The demonstration of more extensive sequence diversity in buffalo-derived parasites than in those maintained in cattle is consistent with the idea that a limited subset of the *T. parva* population has become adapted for maintenance by tick transmission in cattle, or that many parasite genotypes cannot be maintained in the cattle tick transmission cycle. These findings are also consistent with results of vaccination studies, which have provided evidence that immunity induced by infection and treatment with a mixture of parasite isolates is not always effective against challenge with buffalo-derived parasites [47]. Although there was clear evidence of positive selection for amino acid changes in both antigens, these were not significantly enriched within the known CD8^+^ T-cell epitope regions. Additional functional studies of the CD8^+^ T-cell epitopes, currently underway, will identify those alleles that are able to escape CD8^+^ T cell recognition and, by identifying the amino acid substitutions that are critical for escape, may shed further light on whether these antigens are subject to selection by cellular immunity.

## Supporting Information

Figure S1 **Multiple sequence alignment of 35 Tp1 alleles obtained in this study.** The single CD8 T-cell epitope is overlined (plain line). The two polymorphic nucleotides in the epitope domain are shadowed. Positions of flanked residues in the Tp1 gene fragment are numbered. There are 3 size-polymorphic Tp1 of 444, 432 and 408 nucleotides, respectively. The two indels are overlined with a broken line (deletion) and a dotted line (insertion). (*) indicates identical residues. The frequency of each allele is indicated in square brackets, when larger than 1. The flanked PCR primers regions are boxed.
(PDF)

Figure S2 **Multiple sequence alignment of 43 Tp2 alleles obtained in this study.** The frequency of each allele is indicated in square brackets, when larger than 1. Positions of flanked residues in the Tp2 gene fragment are numbered. (*) indicates identical residues. The flanked PCR primers regions are boxed.
(PDF)

Table S1 **Cell lines infected with** ***T. parva*** **cattle-derived parasite stocks isolated from different geographic areas of eastern and southern Africa.** **A**. Laboratory samples. **B**. Kenyan field isolates from cattle with no association with buffalo. **C**. Isolates derived from buffalo.
(DOC)

Table S2 **Tp1 gene alleles and their corresponding antigen variants.**
(DOC)
