# Supplementary material for: Two Theileria parva CD8 T Cell Antigen Genes Are More Variable in Buffalo than Cattle Parasites, but Differ in Pattern of Sequence Diversity
Source: PLoS One. 2011 Apr 29;6(4):e19015. doi: 10.1371/journal.pone.0019015 (PMC3084734; doi:10.1371/journal.pone.0019015)
Supplement: Figure S1 — Multiple sequence alignment of 35 Tp1 alleles obtained in this study. The single CD8 T-cell epitope is overlined (plain line). The two polymorphic nucleotides in the epitope domain are shadowed. Positions of flanked residues in the Tp1 gene fragment are numbered. There are 3 size-polymorphic Tp1 of 444, 432 and 408 nucleotides, respectively. The two indels are overlined with a broken line (deletion) and a dotted line (insertion). (*) indicates identical residues. The frequency of each allele is indicated in square brackets, when larger than 1. The flanked PCR primers regions are boxed. (PDF) [file pone.0019015.s001.pdf]

```
             1                                                                                                            110
Allele-1(24) ATGGCCACTTCAATTGCATTTGCCGCTGATCCTGGATTCTGTTATTTTCTATTAATACCAGGCCCTGACTCGAAACCAATATTCTTCAAAAACGACGGAGATAAATTTTT
Allele-2(11) ATGGCCACTTCAATTGCATTTGCCGCTGATCCTGGATTCTGTTATTTTCTATTAATACCAGGCCCTGACTCGAAACCCATATTCTTCAAAAACGACGGAGATAAATTTTT
Allele-3(2)  ATGGCCACTTCAATTGCATTTGCCGCGGATCCTGGATTCTGTTATTTTCTATTAATACCAGGCCCTGACTCGAAACCCATATTCTTCAAAAACGACGGAGATAAATTTTT
Allele-4(2)  ATGGCCACTTCAATTGCATTTGCCGCTGATCCTGGATTCTGTTATTTTCTATTAATACCAGGCCCTGACTCGAAACCCATATTCTTCAAAAACGACGGAGATAAATTTTT
Allele-5     ATGGCCACTTCAATTGCATTTGCCGCTGATCCTGGATTCTGTTATTTTCTATTAATACCAGGCCCTGACTCGAAACCCATATTCTTCAAAAACGACGGAGATAAATTTTT
Allele-6     ATGGCCACTTCAATTGCATTTGCCGCTGATCCTGGATTCTGTTATTTTCTATTAATACCAGGCCCTGACTCGAAACCCATATTCTTCAAAAACGACGGAGATAAATTTTT
Allele-7     ATGGCCACTTCAATTGCATTTGCCGCTGATCCTGGATTCTGTTATTTTCTATTAATACCAGGCCCTGACTCGAAACCAATATTCTTCAAAAACGACGGAGATAAATTTTT
Allele-8     ATGGCCACTTCAATTGCATTTGCCGCTGATCCTGGATTCTGTTATTTTCTATTAATACCAGGCCCTGACTCGAAACCAATATTCTTCAAAAACGACGGAGATAAATTTTT
Allele-9     ATGGCCACTTCAATTGCATTTGCCGCGGATCCTGGATTCTGTTATTTTCTATTAATACCAGGCCCTGACTCGAAACCAATATTCTTCAAAAACGACGGAGATAAATTTTT
Allele-10    ATGGCCACTTCAATTGCATTTGCCGCTGATCCTGGATTCTGTTATTTTCTATTAATACCAGGCCCTGACTCGAAACCAATATTCTTCAAAAACGACGGAGATAAATTTTT
Allele-11    ATGGCCACTTCAATTGCATTTGCCGCTGATCCTGGATTCTGTTATTTTCTATTAATACCAGGCCCTGACTCGAAACCAATATTCTTCAAAAACGACGGAGATAAATTTTT
Allele-12    ATGGCCACTTCAATTGCATTTGCCGCTGATCCTGGATTCTGTTATTTTCTATTAATACCAGGCCCTGACTCGAAACCAATATTCTTCAAAAACGACGGAGATAAATTTTT
Allele-14(3) ATGGCCACTTCAATTGCATTTGCCGCTGATCCTGGATTCTGTTATTTTCTATTAATACCAGGCCCTGACTCGAAACCTATATTCTTCAAAAACGACGGAGATAAATTTTT
Allele-20    ATGGCCACTTCAATTGCATTTGCCGCTGATCCTGGATTCTGTTATTTTCTATTAATACCAGGCCCTGACTCGAAACCTATATTCTTCAAAAACGACGGAGATAAATTTTT
Allele-22    ATGGCCACTTCAATTGCATTTGCCGCTGATCCTGGATTCTGTTATTTTCTATTAATACCAGGCCCTGACTCGAAACCTATATTCTTCAAAAACGACGGAGATAAATTTTT
Allele-25    ATGGCCACTTCAATTGCATTTGCCGCTGATCCTGGATTCTGTTATTTTCTATTAATACCAGGCCCTGACTCGAAACCAATATTCTTCAAAAACGACGGAGATAAATTTTT
Allele-29    ATGGCCACTTCAATTGCATTTGCCGCTGATCCTGGATTCTGTTATTTTCTATTAATACCAGGCCCTGACTCGAAACCTATATTCTTCAAAAACGACGGAGATAAATTTTT
Allele-32    ATGGCCACTTCAATTGCATTTGCCGCTGATCCTGGATTCTGTTATTTTCTATTAATACCAGGCCCTGACTCGAAACCTATATTCTTCAAAAACGACGGAGATAAATTTTT
Allele-33    ATGGCCACTTCAATTGCATTTGCCGCTGATCCTGGATTCTGTTATTTTCTATTAATACCAGGCCCTGACTCGAAACCTATATTCTTCAAAAACGACGGAGATAAATTTTT
Allele-34    ATGGCCACTTCAATTGCATTTGCCGCTGATCCTGGATTCTGTTATTTTCTATTAATACCAGGCCCTGACTCGAAACCTATATTCTTCAAAAACGACGGAGATAAATTTTT
Allele-13(7) ATGGCCACTTCAATTGCATTTGCCGCTGATCCTGGATTCTGTTATTTTCTATTAATACCAGGCCCTGACTCGAAACCTATATTCTTCAAAAACGACGGAGATAAATTTTT
Allele-15(2) ATGGCCACTTCAATTGCATTTGCCGCTGATCCTGGATTCTGTTATTTTCTATTAATACCAGGCCCTGACTCGAAACCTATATTCTTCAAAAACGACGGAGATAAATTTTT
Allele-16    ATGGCCACTTCAATTGCATTTGCCGCTGATCCTGGATTCTGTTATTTTCTATTAATACCAGGCCCTGACTCGAAACCAATATTCTTCAAAAACGACGGAGATAAATTTTT
Allele-18    ATGGCCACTTCAATTGCATTTGCCGCTGATCCTGGATTCTGTTATTTTCTATTAATACCAGGCCCTGACTCGAAACCTATATTCTTCAAAAACGACGGAGATAAATTTTT
Allele-21    ATGGCCACTTCAATTGCATTTGCCGCTGATCCTGGATTCTGCTATTTTCTATTAATACCAGGCCCTGACTCGAAACCTATATTCTTCAAAAACGACGGAGATAAATTTTT
Allele-23    ATGGCCACTTCAATTGCATTTGCCGCTGATCCTGGATTCTGTTATTTTCTATTAATACCAGGCCCTGACTCGAAACCAATATTCTTCAAAAACGGCGGAGATAAATTTTT
Allele-26    ATGGCCACTTCAATTGCATTTGCCGCTGATCCTGGATTCTGTTATTTTCTATTAATACCAGGCCCTGACTCGAAACCTATATTCTTCAAAAACGACGGAGATAAATTTTT
Allele-27    ATGGCCACTTCAATTGCATTTGCCGCTGATCCTGGATTCTGTTATTTTCTATTAATACCAGGCCCTGACTCGAAACCTATATTCTTCAAAAACGACGGAGATAAATTTTT
Allele-28    ATGGCCACTTCAATTGCATTTGCCGCTGATCCTGGATTCTGTTATTTTCTATTAATACCAGGCCCTGACTCGAAACCTATATTCTTCAAAAACGACGGAGATAAATTTTT
Allele-30    ATGGCCACTTCAATTGCATTTGCCGCTGATCCTGGATTCTGTTATTTTCTATTAATACCAGGCCCTGACTCGAAACCTATATTCTTCAAAAACGACGGAGATAAATTTTT
Allele-31    ATGGCCACTTCAATTGCATTTGCCGCTGATCCTGGATTCTGTTATTTTCTATTAATACCAGGCCCTGACTCGAAACCAATATTCTTCAAAAACGACGGAGATAAATTTTT
Allele-35    ATGGCCACTTCAATTGCATTTGCCGCTGATCCTGGATTCTGTTATTTTCTATTAATACCAGGCCCTGACTCGAAACCTATATTCTTCAAAAACGACGGAGATAAATTTTT
Allele-17    ATGGCCACTTCAATTGCATTTGCCGCTGATCCTGGATTCTGTTATTTTCTATTAATACCAGGCCCTGACTCGAAACCAATATTCCTCAAAAACGACGGAGATAAATTTTT
Allele-19    ATGGCCACTTCAATTGCATTTGCCGCTGATCCTGGATTCTGTTACTTTCTATTAATACCAGGCCCTGACTCGAAACCAATATTCCTCAAAAACGACGGAGATAAATTTTT
Allele-24    ATGGCCACTTCAATTGCATTTGCCGCTGATCCTGGATTCTGTTATTTTCTATTAATACCAGGCCCTGACTCGAAACCAATATTCCTCAAAAACGACGGAGATAAATTTTT
             ************************** ************** ** ******************************** ****** ********* ***************
```

Epitope coding region

```
              111                                                                                                         220
Allele-1(24)  ACGTTGCGTAGGGTATCCAAAGGTTAAAGAAGAAATGCTAGAAATGGCTACAAAATTCAATAGACTACCAAAGGGCGTGGAAATACCTGCACCTCCAGGAGTAAAACCAG
Allele-2(11)  ACGTTGCGTAGGGTATCCAAAGGTTAAAGAAGAAATTATAGAAATGGCTACAAAATTCAATAGACTACCAAAGGGCGTGGAAATACCTGCACCTCCAGGAGTAAAACCAG
Allele-3(2)   ACGTTGCGTAGGGTATCCAAAGGTTAAAGAAGAAATTATAGAAATGGCTACAAAATTCAATAGACTACCAAAGGGCGTGGAAATACCTGCACCTCCAGGAGTAAAACCAG
Allele-4(2)   ACGTTGCGTAGGGTATCCAAAGGTTAAAGAAGAAATTATAGAAATGGCTACAAAATTCAATAGACTACCAAAGGGCGTGGAAATACCTGCACCTCCAGGAGTAAAACCAG
Allele-5      ACGTTGCGTAGGGTATCCAAAGGTTAAAGAAGAAATTATAGAAATGGCTACAAAATTCAATAGACTACCAAAGGGCGTGGAAATACCTGCACCTCCAGGAGTAAAACCAG
Allele-6      ACGTTGCGTAGGGTATCCAAAGGTTAAAGAAGAAATTATAGAAATGGCTACAAAATTCAATAGACTACCAAAGGGCGTGGAAATACCTGCACCTCCAGGAGTAAAACCAG
Allele-7      ACGTTGCGTAGGGTATCCAAAGGTTAAAGAAGAAATTCTAGAAATGGCTACAAAATTCAATAGACTACCAAAGGGCGTGGAAATACCTGCACCTCCAGGAGTAAAACCAG
Allele-8      ACGTTGCGTAGGGTATCCAAAGGTTAAAGAAGAAATTCTAGAAATGGCTACAAAATTCAATAGACTACCAAAGGGCGTGGAAATACCTGCACCTCCAGGAGTAAAACCAG
Allele-9      ACGTTGCGTAGGGTATCCAAAGGTTAAAGAAGAAATGCTAGAAATGGCTACAAAATTCAATAGACTACCAAAGGGCGTGGAAATACCTGCACCTCCAGGAGTAAAACCAG
Allele-10     ACGTTGCGTAGGGTATCCAAAGGTTAAAGAAGAAATGCTAGAAATGGCTACAAAATTCAATAGACTACCAAAGGGCGTGGAAATACCTGCACCTCCAGGAGTAAAACCAG
Allele-11     ACGTTGCGTAGGGTATCCAAAGGTTAAAGAAGAAATGCTAGAAATGGCTACAAAATTCAATAGACTACCAAAGGGCGTGGAAATACCTGCACCTCCAGGAGTAAAACCAG
Allele-12     ACGTTGCGTAGGGTATCCAAAGGTTAAAGAAGAAATGCTAGAAATGGCTACAAAATTCAATAGACTACCAAAGGGCGTGGAAATACCTGCACCTCCAGGAGTAAAACCAG
Allele-14(3)  ACGTTGCGTAGGGTATCCAAAGGTTAAAGAAGAAATTATAGAAATGGCTACAAAATTCAATAGACTACCAAAGGGCGTGGAAATACCTGCACCTCCAGGAGTAAAACCAG
Allele-20     ACGTTGCGTAGGGTATCCAAAGGTTAAAGAAGAAATGCTAGAAATGGCTACAAAATTCAATAGACTACCAAAGGGCATGGAAATACCCGCACCTCCAGGAGTAAAACCAG
Allele-22     ACGTTGCGTAGGGTATCCAAAGGTTAAAGAAGAAATGCTAGAAATGGCTACAAAATTCAATAGACTACCAAAGGGCATGGAAATACCTGCACCTCCAGGAGTAAAACCAG
Allele-25     ACGTTGCGTAGGGTATCCAAAGGTTAAAGAAGAAATGCTAGAAATGGCTACTAAATTCAATAGATTACCAAAGGGCGTGGAAATACCTGCACCTCCAGGAGTAAAACCAG
Allele-29     ACGTTGCGTAGGGTATCCAAAGGTTAAAGAAGAAATGCTAGAAATGGCTACAAAATTCAATAGACTACCAAAGGGCGTGGAAATACCTGCACCTCCAGGAGTAAAACCAG
Allele-32     ACGTTGCGTAGGGTATCCAAAGGTTAAAGAAGAAATGCTAGAAATGGCTACAAAATTCAATAGACTACCAAAGGGCGTGGAAATACCTGCACCTCCAGGAGTAAAACCAG
Allele-33     ACGTTGCGTAGGGTATCCAAAGGTTAAAGAAGAAATGCTAGAAATGGCTACTAAATTCAATAGATTACCAAAGGGCGTGGAAATACCTGCACCTCCAGGAGTAAAACCAG
Allele-34     ACGTTGCGTAGGGTATCCAAAGGTTAAAGAAGAAATGCTAGAAATGGCTACAAAATTCAATAGACTACCAAAGGGCGTGGAAATACCTGCACCTCCAGGAGTAAAACCAG
Allele-13(7)  ACGTTGCGTAGGGTATCCAAAGGTTAAAGAAGAAATGCTAGAAATGGCTACAAAATTCAATAGACTACCAAAGGGCATGGAAATACCTGCACCTCCAGGAGTAAAACCAG
Allele-15(2)  ACGTTGCGTAGGGTATCCAAAGGTTAAAGAAGAAATGCTAGAAATGGCTACAAAATTCAATAGACTACCAAAGGGCATGGAAATACCTTCACCTCCAGGAGTAAAACCAG
Allele-16     ACGTTGCGTAGGGTATCCAAAGGTTAAAGAAGAAATGCTAGAAATGGCTACTAAATTCAATAGATTACCAAAGGGCGTGGAAATACCTGCACCTCCAGGAGTAAAACCAG
Allele-18     ACGTTGCGTAGGGTATCCAAAGGTTAAAGAAGAAATTATAGAAATGGCTACAAAATTCAACAGACTACCAAAGGGCGTGGAAATACCTGCACCTCCAGGAGTAAAACCAG
Allele-21     ACGTTGCGTAGGGTATCCAAAGGTTAAAGAAGAAATGCTAGAAATGGCTACAAAATTCAATAGACTACCAAAGGGCATGGAAATACCTGCACCTCCAGGAGTAAAACCAG
Allele-23     ACGTTGCGTAGGGTATCCAAAGGTTAAAGAAGAAATGCTAGAAATGGCTACAAAATTCAATAGACTACCAAAGGGCATGGAAATACCTGCACCTCCAGGAGTAAAACCAG
Allele-26     ACGTTGCGTAGGGTATCCAAAGGTTAAAGAAGAAATGCTAGAAATGGCTACAAAATTCAATAGACTACCAAAGGGCATGGAAATACCTGCACCTCCAGGAGTAAAACCAG
Allele-27     ACGTTGCGTAGGGTATCCAAAGGTTAAAGAAGAAATGCTAGAAATGGCTACTAAATTCAATAGATTACCAAAGGGCGTGGAAATACCTGCACCTCCAGGAGTAAAACCAG
Allele-28     ACGTTGCGTAGGGTATCCAAAGGTTAAAGAAGAAATGCTAGAAATGGCTACAAAATTCAATAGACTACCAAAGGGCATGGAAATACCTGCACCTCCAGGAGTAAAACCAG
Allele-30     ACGTTGCGTAGGGTATCCAAAGGTTAAAGAAGAAATGATAGAAATGGCTACAAAATTCAATAGACTACCAAAGGGCGTGGAAATACCTGCACCTCCAGGAGTAAAACCAG
Allele-31     ACGTTGCGTAGGGTATCCAAAGGTTAAAGAAGAAATGCTAGAAATGGCTACAAAATTCAATAGACTACCAAAGGGCATGGAAATACCTTCACCTCCAGGAGTAAAACCAG
Allele-35     ACGTTGCGTAGGGTATCCAAAGGTTAAAGAAGAAATGCTAGAAATGGCTACAAAATTCAATAGACTACCAAAGGGCATGGAAATACCTGCACCTCCAGGAGTAAAACCAG
Allele-17     ACGTTGCGTAGGGTATCCAAAGGTTAAAGAAGAAATGCTAGAAATGGCTACAAAATTCAATAGATTACCAAAGGGCGTGGAAATACCTGCACCTCCAGGAGTAAAACCAG
Allele-19     ACGTTGCGTAGGGTATCCAAAGGTTAAAGAAGAAATGCTAGAAATGGCTACAAAATTCAATAGATTACCAAAGGGCGTGGAAATACCTGCACCTCCAGGAGTAAAACCAG
Allele-24     ACGTTGCGTAGGGTATCCAAAGGTTAAAGAAGAAATGCTAGAAATGGCTACAAAATTCAATAGATTACCAAAGGGCGTGGAAATACCTGCACCTCCAGGAGTAAAACCAG
              ************************************  ************* ******** *** *********** **********  *********************
```

```
                                     1st indel                                                   2nd indel
                 221                                                                                          330
Allele-1(24)     AGGCTCCCACACCTACACCAACGACAATAACTCCTTCTGTACCTCCTACTATACCAACGCCAATAACTCCTTCGGCACCTCCT------------ACTACACCACCTACG
Allele-2(11)     AGGCTCCCACACCTACACCAACGACAATAACTCCTTCTGTACCTCCTACTATACCAACGCCAATAACTCCTTCGGCACCTCCT------------ACTACACCACCTACG
Allele-3(2)      AGGCTCCCACACCTACACCAACGACAATAACTCCTTCTGTACCTCCTACTATACCAACGCCAATAACTCCTTCGGCACCTCCT------------ACTACACCACCTACG
Allele-4(2)      AGGCTCCCACACCTACACCAACGACAATAACTCCTTCTGTACCTCCTACTATACCAACGCCAATAACTCCTTCGGCACCTCCT------------ACTACACCACCTACG
Allele-5         AGGCTCCCACACCTACACCAACGACAATAACTCCTTCTGTACCTCCTACTATACCAACGCCAATAACTCCTTCGGCACCTCCT------------ACTACACCACCTACG
Allele-6         AGGCTCCCACACCTACACCAACGACAATAACTCCTTCTGTACCTCCTACTATACCAACGCCAATAACTCCTTCGGCACCTCCT------------ACTACACCACCTACG
Allele-7         AGGCTCCCACACCTACACCAACGACAATAACTCCTTCTGTACCTCCTACTATACCAACGCCAATAACTCCTTCGGCACCTCCT------------ACTACACCACCTACG
Allele-8         AGGCTCCCACACCTACACCAACGACAATAACTCCTTCTGTACCTCCTACTATACCAACGCCAATAACTCCTTCGGCACCTCCT------------ACTACACCACCTACG
Allele-9         AGGCTCCCACACCTACACCAACGACAATAACTCCTTCTGTACCTCCTACTATACCAACGCCAATAACTCCTTCGGCACCTCCT------------ACTACACCACCTACG
Allele-10        AGGCTCCCACACCTACACCAACGACAATAACTCCTTCTGTACCTCCTACTATACCAACGCCAATAACTCCTTCGGCACCTCCT------------ACTACACCACCTACG
Allele-11        AGGCTCCCACACCTACACCAACGACAATAACTCCTTCTGTACCTCCTACTATACCAACGCCAATAACTCCTTCGGCACCTCCT------------ACTACACCACCTACG
Allele-12        AGGCTCCCACACCTACACCAACGACAATAACTCCTTCTGTACCTCCTACTATACCAACGCCAATAACTCCTTCGGCACCTCCT------------ACTACACCACCTACG
Allele-14(3)     AGGCTCCCACACCTACACCAACGACAATAACTCCTTCTGTACCTCCTACTATACCAACGCCAATAACTCCTTCGGCACCTCCT------------ACTACACCAACTACG
Allele-20        AGGCTCCCACACCTACACCAACGACAATAACTCCCTCTGTACCTCCTACTATACCAACTCCAATAACTCCTTCTGCACCTCCT------------ACTACACCACCTACG
Allele-22        AGGCTCCCACACCTACACCAACGACAATAACTCCCTCTGTACCTCCTACTATACCAACTCCAATAACTCCTTCTGCACCTCCT------------ACTACACCACCTACG
Allele-25        AGGCTCCCACACCTATACCAACGACAATAACTCCCTCTGTACCTCCTACTATACCAACTCCAATAACTCCTTCGGCACCTCCT------------ACTACACCACCTACG
Allele-29        AGGCTCCCACACCTACACCAACGACAATAACTCCCTCTGTACCTCCTACTATACCAACTCCAATAACTCCTTCTGCACCTCCT------------ACTACACCACCTACG
Allele-32        AGGCTCCCACACCTACACCAACGACAATAACTCCCTCTGTACCTCCTACTATACCAACTCCAATAACTCCTTCTGCACCTCCT------------ACTACACCACCTACG
Allele-33        AGGCTCCCACACCTACACCAACGACAATAACTCCTTCTGCACCTCCTACTATACCAACTCCAATAACTCCTTCTACACCTCCT------------ACTACACCACCTAAG
Allele-34        AGGCTCCCACACCTACACCAACGACAATAACTCCCTCTGTACCTCCTACTATACCAACTCCAATAACTCCTTCTGCACCTCCT------------ACTACACCACCTACG
Allele-13(7)     AGGCTCCCACACCTACACCAACGACAATAACTCCTTCTGTACCTCCTACTATACCAACTCCAATAACTCCTTCTGCACCTCCTTCTGCACCTCCTACTACACCACCTAAG
Allele-15(2)     AGGCTCCCACACCTACACCAACGACAATAACTCCCTCTGTACCTCCTACTATACCAACTCCAATAACTCCTTCGGCACCTCCTACTACACCACCTACTACACCACCTACG
Allele-16        AGGCTCCCACACCTACACCAACGACAATAACTCCTTCTGCACCTCCTACTATACCAACTCCAATAACTCCTTCTGCACCTCCTACTACACCTCCTACTACACCACCTAAG
Allele-18        AGGCTCCCACACCTACACCAACGACAATAACTCCTTCTGCACCTCCTACTATACCAACTCCAATAACTCCTTCTGCACCTCCTACTACACCTCCTACTACACCACCTACG
Allele-21        AGGCTCCCACACCTACACCAACGACAATAACTCCTTCTGTACCTCCTACTATACCAACGCCAATAACTCCTTCTGCACCTCCTTCTGCACCTCCTACTACACCACCTACG
Allele-23        AGGCTCCCACACCTACACCAACGACAATAACTCCTTCTGTACCTCCTACTATATCAACTCCAATAACTCCTTCTGCACCTCCTTCTGCACCTCCTACTATACCACCTAAG
Allele-26        AGGCTCCCACACCTACACCAACGACAATAACTCCTTCTGTACCTCCTACTATACCAACTCCAATAACTCCTTCTGCACCTCCTTCTGCACCTCCTACTACACCACCTACG
Allele-27        AGGCTCCCACACCTACACCAACGACAATAACTCCTTCTGCACCTCCTACTATACCAACTCCAATAACTCCTTCTGCACCTCCTACTACACCTCCTACTACACCACCTAAG
Allele-28        AGGCTCCCACACCTACACCAACGACAATAACTCCCTCTGTACCTCCTACTATACCAACTCCAATAACTCCTTCGGCACCTCCTACTACACCACCTACTACACCACCTACG
Allele-30        AGGCTCCCACACCTACACCAACGACAATAACTCCTTCTGTACCTCCTACTATACCAACTCCAATAACTCCTTCTGCACCTCCTACTACACCTCCTACTACACCACCTAAG
Allele-31        AGGCTCCCACACCTACACCAACGACAATAACTCCCTCTGTACCTCCTACTATACCAACTCCAATAACTCCTTCGGCACCTCCTACTACACCACCTACTACACCACCTACG
Allele-35        AGGCTCCCACACCTACACCAACGACAATAACTCCTTCTGTACCTCCTACTATACCAACTCCAATAACTCCTTCTGCACCTCCTACTGCACCTCCTACTACACCACCTAAG
Allele-17        AGGCTCCCACACCTACACCA------------------------------------ACTCCAATAACTCCTTCGGCACCTCCTACTACACCACCTACTACACCACCTACG
Allele-19        AGGCTCCCACACCTACACCA------------------------------------ACTCCAATAACTCCTTCTGCACCTCCTACTACACCTCCTACTACACCACCTAAG
Allele-24        AGGCTCCCACACCTACACCA------------------------------------ACTCCAATAACTCCTTCTGCACCTCCTACTACACCTCCTACTACACCACCTAAG
                 *************** ****                                    ** **************  ********            **** **** *** *
```

```
                  331                                                                                                444
Allele-1(24)      GGACTAAATTTTAACTTGACAGTTCAGAACAAATTCATGATAGGTTCGCAAGAAGTTAAGTTAAATATAACTCACGAATACGAGGGTGTATACGAAGCTCATAAATATTTCATTTAA
Allele-2(11)      GGACTAAATTTTAACTTGACAGTTCAGAACAAATTCATGGTAGGTTCGCAAGAAGTTAAGTTAAATATAACTCACGAATACGATGGTGTATACGAAGCTCATAAATATTTCATTTAA
Allele-3(2)       GGACTAAATTTTAACTTGACAGTTCAGAACAAATTCATGGTAGGTTCGCAAGAAGTTAAGTTAAATATAACTCACGAATACGATGGTGTATACGAAGCTCATAAATATTTCATTTAA
Allele-4(2)       GGACTAAATTTTAACTTGACAGTTCAGAACAAATTCATGGTAGGTTCGCAAGAAGTTAAGTTAAATATAACTCACGAATACGAGGGTGTATACGAAGCTCATAAATATTTCATTTAA
Allele-5          GGACTAAATTTTAACTTGACAGTTCAGAACAAATTCATGGTAGGTTCGCAAGAAGTTAAGTTAAATATACCTCACGAATACGATGGTGTATACGAAGCTCATAAATATTTCATTTAA
Allele-6          GGACTAAATTTTAACTTGACAGTTCAGAACAAATTCATGATAGGTTCGCAAGAAGTTAAGTTAAATATAACTCACGAATACGATGGTGTATACGAAGCTCATAAATATTTCATTTAA
Allele-7          GGACTAAATTTTAACTTGACAGTTCAGAACAAATTCATGATAGGTTCGCAAGAAGTTAAGTTAAATATAACTCACGAATACGAGGGTGTATACGAAGCTCATAAATATTTCATTTAA
Allele-8          GGACTAAATTTTAACTTGACAGTTCAGAACAAATTCATGGTAGGTTCGCAAGAAGTTAAGTTAAATATAACTCACGAATACGATGGTGTATACGAAGCTCATAAATATTTCATTTAA
Allele-9          GGACTAAATTTTAACTTGACAGTTCAGAACAAATTCATGATAGGTTCGCAAGAAGTTAAGTTAAATATAACTCACGAATACGAGGGTGTATACGAAGCTCATAAATATTTCATTTAA
Allele-10         GGACTAAATTTTAACTTGACAGTTCAGAACAAATTCATGATAGGTTCGCCAGAAGTTAAGTTAAATATAACTCACGAATACGAGGGTGTATACGAAGCTCATAAATATTTCATTTAA
Allele-11         GGACTAAATTTTAACTTGACAGTTCAGAACAAATTCATGATAGGTTCGCAAGAAGTTAATTTAAATATAACTCACGAATACGAGGGTGTATACGAAGCTCATAAATATTTCATTTAA
Allele-12         GGACTAAATTTTAACTTGACAGTTCAGAACAAATTCATGATAGGTTCGCAAGAAGTTAAGTTTAAATTTACTCACGAATACGAGGGTGTATACGAAGCTCATAAATATTTCATTTAA
Allele-14(3)      GGACTAAATTTTAACTTGACACGTCAGAACAAATTCATGATAGGTTCGCAAGAAGTTAAGTTAAATATAACTTACGAATACGATGGTGTATACGAAGCTCATAAATATTTCATTTAA
Allele-20         GGACTAAATTTTAACTTGATAGTTCAGAACAAATTCATGGTAGGTTCGCAAGAAGTTAAGTTAAGTATAACTTACGAATACGATGGTGAATACGAAGCTCATAAATATTTCATTTAA
Allele-22         GGACTAAATTTTAACTTGATAGTTCAGAACAAATTCATGGTAGGTTCGCAAGAAGTTAAGTTAAGTATAACTTACGAATACGATGGTGAATACGAAGCTCATAAATATTTCATTTAA
Allele-25         GGACTAAATTTTAACTTGACAGTTCAGAACAAATTCATGGTAGGCTCGCAAGAAGTTAAGTTAAGTATAACTTACGAATACGATGGTGTATACGAAGCTCATAAATATTTCATTTAA
Allele-29         GGACTAAATTTTAACTTGATAGTTCAGAACAAATTCATGGTAGGTTCGCAAGAAGTTAAGTTAAGTATAACTTACGAATACGATGGTGAATACGAAGCTCATAAATATTTCATTTAA
Allele-32         GGACTAAATTTTAACTTGACACTTCAGAACAAATTCATGATAGGTTCGCAAGAAGTTAAGTTAAGTATAACTTACGAATACGATGGTGAATACGAAGCTCATAAATATTTCATTTAA
Allele-33         GGACTAAATTTTAACTTGACACTTCAGAACAAATTCATGGTAGGTTCGCAAGAAGTTAAGTTAAATATAACTCACGAATACGAGGGTGTATACGAAGCTCATAAATATTTCATTTAA
Allele-34         GGACTAAATTTTAACTTGATAGTTCACAACAAATTCATGGTAGGTTCGCAAGAAGTTAAGTTAAATATAACTTACGAATACGATGGTGAATACGAAGCTCATAAATATTTCATTTAA
Allele-13(7)      GGACTAAATTTTAACTTGACACTTCAGAACAAATTCATGATAGGTTCGCAAGAAGTTAAGTTAAGTATAACTCACGAATACGATGGTGTATACGAAGCTCATAAATATTTCATTTAA
Allele-15(2)      GGACTAAATTTTAACTTGACAGTTCAGAACAAATTCATGGTAGGCTCGCAAGAAGTTAAGTTAAGTATAACTTACGAATACGATGGTGTATACGAAGCTCATAAATATTTCATTTAA
Allele-16         GGACTAAATTTTAACTTGACACTTCAGAACAAATTCATGGTAGGTTCGCAAGAAGTTAAGTTAAATATAACTCACGAATACGAGGGTGTATACGAAGCTCATAAATATTTCATTTAA
Allele-18         GGACTAAATTTTAACTTGACACTTCAGAACAAATTCATGGTAGGTTCGCAAGAAGTTAAGTTAAGTATAACTTACGAATACGATGGTGTATACGAAGCTCATAAATATTTCATTTAA
Allele-21         GGACTAAATTTTAACTTGACACTTCAGAACAAATTCATGATAGGTTCGCAAGAAGTTAAGTTAAGTATAACTCACGAATACGAGGGTGTATACGAAGCTCATAAATATTTCATTTAA
Allele-23         GGACTAAATTTTAACTTGACACTTCAGAACAAATTCATGATAGGTTCGCAAGAAGGTAAGTTAAGTATAACTCACGAATACGATGGTGTATTTGAAGCTCATAAATATTTCATTTAA
Allele-26         GGACTAAATTTTAACTTGACACTTCAGAACAAATTCATGATAGGTTCGCAAGAAGTTAAGTTAAGTATAACTCACGAATACGATGGTGTATACGAAGCTCATAAATATTTCATTTAA
Allele-27         GGACTAAATTTTAACTTGACACTTCAGAACAAATTCATGGTAGGTTCGCAAGAAGTTAAGTTAAATATAACTCACGAATACGAGGGTGTATACGAAGCTCATAAATATTTCATTTAA
Allele-28         GGACTAAATTTTAACTTGACAGTTCAGAACAAATTCATGGTAGGCTCGCAAGAAGTTAAGTTAAGTATAACTTACGAATACGATGGTGTATACGAAGCTCATAAATATTTCATTTAA
Allele-30         GGACTAAATTTTAACTTGACAGTTCAGAACAAATTCATGGTAGGTTCGCAAGAAGTTAAGTTAAGTATAACTTACGAATACGATGGTGTATACGAAGCTCATAAATATTTCATTTAA
Allele-31         GGACTAAATTTTAACTTGACAGTTCAGAACAAATTCATGGTAGGCTCGCAAGAAGTTAAGTTAAGTATAACTTACGAATACGATGGTGTATACGAAGCTCATAAATATTTCATTTAA
Allele-35         GGACTAAATTTTAACTTGACACTTCAGAACAAATTCATGATAGGTTCGCAAGAAGTTAAGTTAAGTATAACTCACGAATACGATGGTGTATACGAAGCTCATAAATATTTCATTTAA
Allele-17         GGACTAAATTTTAACTTGACAGTTCAGAACAAATTCATGGTAGGCTCGCAAGAAGTTAAGTTAAGTATAACTTACGAATACGATGGTGTATATGAAGCTCATAAATATTTCATTTAA
Allele-19         GGACTAAATTTTAACTTGACAGTTCAGAACAAATTCATGGTAGGTTCGCAAGAAGTTAAGTTAAATATAACTCACGAATACGAGGGTGTATACGAAGCTCATAAATATTTCATTTAA
Allele-24         GGACTAAATTTTAACTTGACACTTCAGAACAAATTCATGATAGGTTCGCAAGAAGTTAAGTTAAGTATAACTCACGAATACGATGGTGTATACGAAGCTCATAAATATTTCATTTAA
                  ******************* *  *** ************ **** **** ***** *** ** *   *  ** ********** **** **  ************************
```

Figure S1. Multiple sequence alignment of 35 Tp1 alleles obtained in this study
