# Supplementary material for: Two Theileria parva CD8 T Cell Antigen Genes Are More Variable in Buffalo than Cattle Parasites, but Differ in Pattern of Sequence Diversity
Source: PLoS One. 2011 Apr 29;6(4):e19015. doi: 10.1371/journal.pone.0019015 (PMC3084734; doi:10.1371/journal.pone.0019015)
Supplement: Figure S2 — Multiple sequence alignment of 43 Tp2 alleles obtained in this study. The frequency of each allele is indicated in square brackets, when larger than 1. Positions of flanked residues in the Tp2 gene fragment are numbered. (*) indicates identical residues. The flanked PCR primers regions are boxed. (PDF) [file pone.0019015.s002.pdf]

```
                    1                                                                                                                    120
Allele-1[24]        ATGAAATTGGCCGCCAGATTAATTAGCCTTTACTTTATTATTTACATTTTACATTCCCCAGTGCTGGGAGGTAATTGTAGTCATGAAGAACTAAAAAAATTGGGAATGCTAGAGGGCGAT
Allele-2[8]         ATGAAATTGGCCGCCAGATTAATTAGCCTTTACTTTATTATTTTCATTCTACCTTCCTCAGTCTTGGGAGGTAATTGTAGTGACGATGAACTGGATACTTTGGGTATGTTGGACAAACCA
Allele-3[4]         ATGAAATTGGCCGCCAGATTAATTAGCCTTTACTTTATTATTTACATTTTACATTCCCCAGTGCTGGGAGGTAATTGTAGTGATGAAGAACTAAATAAATTGGGAATGCTAGAGGGCGAT
Allele-4[2]         ATGAAATTGGCCGCCAGATTAATTAGCCTTTACTTTATTATTTTCATTCTACCTTCCTCAGTCTTGGGAGGTAATTGTAGTGACAATGAGCTGGATACTTTGGGTTTGTTGGACAAACCA
Allele-5            ATGAAATTGGCCGCCAGATTAATTAGCCTTTACTTTATTATTTACATTTTACATTCCCCAGTGCTGGGAGGTAATTGTAGTCATGAAGAACTAAAAAAATTGGGAATGCTAGAGGGCGAT
Allele-6[3]         ATGAAATTGGCCGCCAGATTAATTAGCCTTTACTTTATTATTTTCATTCTACCTTCCTCAGTCTTGGGAGGCAATTGTAGTGACGAAGAACTAAAAAAATTGGGAATGTTAAAGGGCGAT
Allele-7[2]         ATGAAATTGGCCGCCAGATTAATTAGCCTTTACTTTATTATTTTCATTCTACCTTCCTCAGTCTTGGGAGGTAATTGTAGTGATGAGGAACTAGAAAGTTTGGGTATGTTGGACAAACCG
Allele-8            ATGAAATTGGCCGCCAGATTAATTAGCCTTTACTTTATTATTTTCATTCTACATTCCCCAGTGCTGGGAGGTAATTGTAGTGATGAAGAACTAAAAAAAATGGGAATGTTAAAGGGCGAT
Allele-9            ATGAAATTGGCCGCCAGATTAATTAGCCTTTACTTTATTATTTTCATTCTACCTTCCCCAGTGTTGGGAGGTAATTGTAGTAATGAAGAACTAAAAAAATTGGGAATGGTAGAGGGCGAA
Allele-10           ATGAAATTGGCCGCCAGATTAATTAGCCTTTACTTTATTATTTTCATTCTACATTCCCCAGTGCTGGGAGGCAATTGTAGTGACGAAGAACTAAAAAAATTGGGAATGTTAAAGGGCGAT
Allele-11           ATGAAATTGGCCGCCAGATTAATTAGCCTTTACTTTATTATTTACATTTTACATTCCCCAGTGCTGGGAGGTAATTGTAGTCATGAAGAACTAAAAAAATTGGGAATGCTAGAGGGCGAT
Allele-12           ATGAAATTGGCCGCCAGATTAATCAGTCTTTACTTTATTATTTTCATTCTACCTTCCTCAGTGCTGGGAGGTAATTGTAGTAATGAAGAACTAAAAAAATTGGGAATGGTAGAGGCCGAA
Allele-13           ATGAAATTGGCCGCCAGATTAATCAGCCTTTACTTTATTATTTTCATTCTACCTTCCTCAGTCTTGGGAGGTAATTGTAGTGACGATGAACTAAAGAAAATGGGTTTGATAGAAACCCCT
Allele-14           ATGAAATTGGCCGCCAGATTAATTAGCCTTTGCTTTATTATTTACATTTTACATTCCCCAGTGCTTGGAGGTAATTGTAGTCATGAAGAACTAAAAAAATTGGGAATGCTAGAGGGCGAT
Allele-15           ATGAAATTGGCCGCCAGATTAATTAGCCTTTACTTTATTATTTTCATTCTACCTTCCTCAGTCTTGGGAGGTAATTGTAGTGATGAAGAACTAAAAAAAATGGGAATGTTAAAGGGCGAT
Allele-16           ATGAAATTGGCCGCCAGATTAATTAGCCTTTACTTTATTATTTACATTTTACATTCCCCAGTGCTGGGAGGTAATTGTAGTCATAGTGAGCTAGAAACTTTGGGTATGTTGGACAAACCG
Allele-17           ATGAAATTGGCCGCCAGATTAATTAGCCTTTACTTTATTATTTTCATTCTACCTTCCTCAGTCTTGGGAGGTAATTGTAGTGATGAGGAACTAAAAAAATTGGGAATGCTAGAGGGCGAT
Allele-18           ATGAAATTGGCCGCCAGATTAATTAGCCTTTACTTTATTATTTTCATTCTACCTTCCAAGATCTTGCGGGATCAATGTACTCCTCCAGAACTTGAGGCACTGGGAAGGTTGCTCAACTCG
Allele-19           ATGAAATTGGCCGCCAGATTAATTAGCCTTTACTTTATTATTTACATTTTACATTCACCAGTGCTGGGAGGTAATTGTAGTCATGAAGAACTAAAAAAAATGGGAATGTTGGTCAAACCG
Allele-20           ATGAAATTGGCCGCCAGATTAATTAGCCTTTACTTTATTATTTACATTTTACATTCACCAGTGCTGGGAGGTAATTGTAGTCATGAAGAACTAAAAAAATTGGGAATGCTAGAGGGCGAT
Allele-21           ATGAAATTGGCCGCCAGATTAATTAGCCTTTACTTTATTATTTACATTCTACATCCTACAGTCTTGGGAGGTAATTGTACTGAGGAAGAACTAAAAAGAATGGGAATGTTGGTCAAACCG
Allele-22           ATGAAATTGGCCGCCAGATTAATTAGCCTTTACTTTATTATTTACATTTTACATTCCCCAGTGCTGGGAGGTAATTGTACTAGTGAAGAACTAAAAAAATTGGGAATGGTAGAGGGTGAA
Allele-23           ATGAAATTGGCCGCCAGATTAATTAGCCTTTACTTTATTATTTTCATTCTACCTTCCTCAGTCTTGGGAGGTAATTGTAGTGATGATGAACTAAGAAAATTGGGAATGTTAAAGGGCGAT
Allele-24           ATGAAATTGGCCGCCAGATTAAAAAGCCTTTACTTTATTATTTTCATTCTACCTTCCTCAGTCTTGGGAGGTAATTGTAGTGACGATGAACTAAAGAAAATGGGTTTGATAGAAACCCCT
Allele-25           ATGAAATTGGCCGCCAGATTAATTAGCCTTTACTTTATTATTTACATTTTACATTCCCCAGTGCTGGGAGGTAATTGTAGTGATGAAGAACTAAAAAAATTGGGAATGCTAGAGGGCGAT
Allele-26           ATGAAATTGGCCGCCAGATTAATTAGTCTTTACTTTGTTATTTTCATTCTACCTTCCTCAGTCTTGGGAGGTAATTGTACTAATGAAGAACTAAAAAAATTGGGAATGGTAGTGGGTGAA
Allele-27           ATGAAATTGGCCGCCAGATTAATTAGCCTTTACTTTATTATTTTCATTCTACCTTCCTCAGTCTTGGGAGGCAATTGTAATGATGATGAACTAAAAAGAATGGGAATGGTAGAGGGCGAT
Allele-28           ATGAAATTGGCCGCCAGATTAATTAGCCTTTACTTTATTATTTTCATTCTACCTTCCTCAGTCTTGGGAGGCAATTGTAGTGACGAAGAACTAAAAAAATTGGGAATGTTAAAGGGCGAT
Allele-29           ATGAAATTGGCCGCCAGATTAATTAGCCTTTACTTTATTATTTTCATTCTACCTTCCTCAGTCTTGGGAGGTAATTGTAGTGATGATGAACTAAGAAAATTGGGAATGTTAAAGGCCGAT
Allele-30           ATGAAATTGGCCGCCAGATTAATTAGCCTTTACTTTATTATTTTCATTCTACCTTCCTCAGTCTTGGGAGGTAATTGTAGTGATGAAGAGCTAAAAGAATTGGGAATGCTAGACGGTGAT
Allele-31           ATGAAATTGGCCGCCAGATTAATTAGCCTTTACTTTATTATTTTCATTCTACCTTCCTCAGTCTTGGGAGGTAATTGTACTGAGGATGAACTAAAAAAATTGGGAATGGTAGGAGGTGAT
Allele-32           ATGAAATTGGCCGCCAGATTAATTAGCCTTTACTTTATTATTTTCATTCTACCTTCCTCAGTCTTGGGAGGCAATTGTAATGATGAGGAACTAGAAAATTTGGGTATGTTGGACAAACCG
Allele-33           ATGAAATTGGCCGCCAGATTAATTAGCCTTTACTTTATTATTTACATTTTACATTCCCCAGTGCTGGGAGGTAATTGTAGTGATGAAGAACTAAAAAAATTGGGAATGCTAGAGGGCGAT
Allele-34           ATGAAATTGGCCGCCAGATTAATTAGCCTTTACTTTATTATTTTCATTCTACCTTCCTCAGTCTTGGGAGGTAATTGTACTGTGGAAGAACTAAGAGAAATGGGAATGGTAGAAGGTGAT
Allele-35           ATGAAATTGGCCGCCAGATTAATAAGGCTTTACTTTATTATTTTCATTCTACATTCTTCAGTCTTGGGAAATAATTGTAGTGATGAGGAATTAAACAAATTGGGAATGTTAGACGGTCCT
Allele-36           ATGAAATTGGCCGCCAGATTAATTAGCCTTTACTTTATTATTTACATTTTACATTCCCCAGTGCTGGGAGGTAATTGTAGTGATGAAGAACTAAAAAAATTGGGAATGCTAGAGGGCGAT
Allele-37           ATGAAATTGGCCGCCAGATTAATTAGCCTTTACTTTGTTATTTTCATTCTACCTTCCTCAGTCTTGGGAGGTAATTGTAGTGATGATGAACTAAATAAATTGGGAATGTTGGTCAAACAG
Allele-38           ATGAAATTGGCCGCCAGATTAATTAGCCTTTACTTTATTATTTACATTTTACATTCCCCAGTGCTGGGAGGTAATTGTAGTCATGAAGAACTAAAAAAATTGGGAATGCTAGAGGGCGAT
Allele-39           ATGAAATTGGCCGCCAGATTAATTAGCCTTTACTTTATTATTTTCATCCTACCTTCCTCAGTCTTGGGAGGCAATTGTACTGATGAGGAACTAGAAAATTTGGGTATGTTGGACAAACCG
Allele-40           ATGAAATTGGCCGCCAGATTAATTAGCCTTTACTTCATTATTTTCATTCTACCTTCTTCGGTCTTTGGAGGTAATTGTACTGAAGATGAACTAAAGAATATGGGTTTGATAGAAACCCCT
Allele-41           ATGAAATTGGCCGCCAGATTAATTAGCCTTTACTTTATTATTTTCATTCTACCTTCCTCAGTCTTGGGAGGCAATTGTAGTGACGAAGAACTAAAAAAATTGGGAATGTTAAAGGGCGAT
Allele-42           ATGAAATTGGCCGCCAGATTAATTAGCCTTTACTTTATTATTTTCATTCTACCTTCCTCAGTCTGTGGAGGCAATGGTAGTGACGAAGACCTAAAAAAATTGGGAATGTTAAAGGGCGAT
Allele-43           ATGAAATTGGCCGCCAGATTAATTAGTCTTTACTTTGTTATTTTCATTCTACCTTCCTCAGTCTTGGGAGGTAATTGTACTAATGAAGAACTGAAAAAATTGGGAATGGTAGTGGGTGAA
                    **********************  ** **** ***  ****** ***  *** * *     *     *     *  *** *      **  *        ****   * *
```

	121 240
Allele-1[24]	GGTTTCGACAGGGATGCATTGTTCAAATCATCACATGGTATGGGAAAGGTAGGAAAAAGGTATGGTCTTAAAACTACTCCAAAAGTAGATAAAGTCTTAGCAGATCTTGAAACACTGTTT
Allele-2[8]	GATCTTGACAAGAATAGATTGTTCTTAACATCACATGGTATGGGAAGGATAGGGAGAAGGTATGGTATTAGACCTGGAACAAAAACAGAAAAATTCTTAAAAGAACTTAAAAAATTATTT
Allele-3[4]	GGTTTCGACAGGGATGCATTGTTCAAATCATCACATGGTATGGGAAAGGTAGGAAAAAGGTATGGTCTTAAAACTACTCCAAAAGTAGATAAAGTCTTAGCAGATCTTGAAACACTGTTT
Allele-4[2]	GATCTTGACAAGAATAGATTGTTTTTAACATCACATGGAATGGGAAAGATAGGTAGAAGGTTTGGTATTAGACCTGGAACAAAAACAGAAAAATTCTTAAAAGAACTTACTAAATTATTT
Allele-5	GGTTTCGACAGGGATGCATTGTTCAAATCATCACATGGTATGGGAAAGGTAGGAAAAAGGTATGGTCTTAAAACTACTCCAAAAGTAGATAAAGTCTTAGCAGATCTTGAAACACTGTTT
Allele-6[3]	GGTTTCGAAAAGGAAAGATTGTTCAAAACATCACATAGTATGGGAATGATAGGGAAAAGGCATGCTCTTAAAACAAGTCCAAAAATAGATAAAGTCGTAGTAGATCTTGAAACACTGTTT
Allele-7[2]	GACCCTGTCAAGGAAAGATTGTTCTTAACATCTAAGGCTATGACTACAGTAGGGAAAAGGCATGGTCTTAGACCTGGAACTCCAAAGGAAAAATTTCAAAGAGAACTTACTAAACTTCTT
Allele-8	GGTTTCGAAAAGGAAAGATTGTTCAGATCATCACATGGAATGGGAAAGGTAGGTAAAATGCATGGTCTTAAATCAACTCCAAAAGTGGATAAAGTCTTAGAAGATCTTGAAACACTGTTT
Allele-9	GGTTTAGACAAGGATGCATTGTTCAAATCATCTAAGGGTATGGGAAAGGTAGGGAAAAGGTATGGTATTAAATCTAGTCCAAAAAAGGATAAAATCTTTGAAGATCTTGAAACACTTCTT
Allele-10	GGTTTCGAAAAGGAAAGATTGTTCAAAACATCACATAGTATGGGAATGATAGGGAAAAGGCATGCTCTTAAAACAAGTCCAAAAATAGATAAAGCTCTCGTAGATCTTGAAACACTGTTT
Allele-11	GGTTTCGACAGGGATGCATTGTTCAAATCATCACATGGTATGGGAGAGGTAGGAAAAAGGTATGGTCTTAAAACTACTCCAAAAGTAGATAAAGTCTTAGCAGATCTTGAAACACTGTTT
Allele-12	GGTTTCGATAAGGAGGCATTGTTCAAATCATCAAAGGGTATGACAAAAGTAGGAAAAAAGTATGGTATTAGACCTGGAACAACAAAAGATAAACTTTCTAGAGATCTTGGTAAACTTCTT
Allele-13	GGTTTCGATAAGGAAGTATTGTTTAAATCATCAAAGGCTATGACGACAACAGGAAAAAAGCATGGAATTAGACTTGGAGCAACAAAAGAGAAACTTGCTAATGAACTTGGTAAACTTCTT
Allele-14	GGTTTCGACAGGGATGCATTGTTCAAATCATCACATGGTATGGGAAAGGTAGGAAAAAGGTATGGTCTTAAAACTACTCCAAAAGTAGATAAAGTCTTAGCAGATCTTGAAACACTGTTT
Allele-15	GGTTTCGAAAAGGAAAGATTGTTCAGATCATCACATGGAATGGGAAAGGTAGGTAAAATGCATGGTCTTAAATCAACTCCAAAAGTGGATAAAGTCTTAGAAGATCTTGAAACACTGTTT
Allele-16	GATCCTGACAAGCAAAGATTGTTCTTAACATCTAAGGCTATGTCGACAGTAGGGAAAAAATATGGTATTAGACCTGGAACAAAAACAGAAAAATTTCTAAAAGAACTTACTACTCTTCTT
Allele-17	GGTTTCGACAGGGATGCATTGTTCAAATCATCACATGGCATGGGAAAGGTAGGAAAAAGGTATGGTCTTAAAACTACTCCCAAAGTAGATAAAGTTCTAGCAGATCTTGAAACACTGCTT
Allele-18	GATCCTGACAGGGAAACATTGTTTAAAACATCAAAGGCTATGACTATGACAGGGAGAAAGCATGGTATTAGACCTGGAACTTCAAAAGAGAAACTTACTAGAGAACTTACTAAACTTCTT
Allele-19	GATCCTGACAAGGAAACATTGTTCAAAACATCAAAGGCTATGACTATGACAGGGAGAAAGCATGGTATTAGACCTGGAACTTCAAAAGAGAAACTTACTAGAGAGCCTCCTGTTCTTCTT
Allele-20	GGTTTCGACAGGGATGCATTGTTCAAATCATCACATGGTATGGGAAAGGTAGGAAAAAGGTATGGTCTTAAAACTACTCCAAAAGTAGATAAAGTCTTAGCAGATCTTGAAACACTGTTT
Allele-21	GATCCTGACAAGGAAACATTGTTCAAAACATCAAAGGCTATGACTATGACAGGGAGAAAGCATGGTATTAGACCTGGAACTTCAAAAGAGAAACTTACTAGAGAACTTACTAAACTTCTT
Allele-22	GGTTTCGACAGGGAGGCATTGTTCAAAACATCTAATGGTATGACAAAGGTAGGAAAAAAGTATGGTCTTAAACCTACTCCTACAAAAGATAAACTTACTAAAGAACTTGCTACACTTCTT
Allele-23	GGTTTCGACAGGGATGCATTGTTCAAATCATCACATGGAATGGGAAAGGTAGGTAGAATGCATGGTCTTAAAACGACTCCAAAAGTAGAGAGAGTCTTAAAAGATCTTGAAACACTGTTT
Allele-24	GGTTTCGATAAGGAAGTATTGTTTAAATCATCAAAGGCTATGACGACAACAGGAAAAAAGCATGGAATTAGACTTGGAGCAACAAAAGAGAAACTTGCTAATGAACTTGGTAAACTTCTT
Allele-25	GGTTTCGACAGGGATGCATTGTTCAAATCATCACATGGTATGGGAAAGGTAGGAAAAAGGTATGGTCTTAAAACTACTCCAAAAGTAGATAAAGTCTTAGCAGATCTTGAAACACTGTTT
Allele-26	GGTTTAGATATGGAGGCATTGTTCAAAACATCTAAGGGTATGACGAAAGTAGGGAGAAAGTATGGTATTAGACCTGGAACTACAAAAGATAAACTTACTAGAGAGCTTACTAGACTTCTT
Allele-27	GGTTTCGATAAGGAATTATTGTTTAAATCATCAAAGGGCATGACGGCAGTAGGGAAAAAGTATGGTATTAGACCAGGAATATCGGTAGATAAATTCCTAAGTGAACTTCAAAAATTATTT
Allele-28	GGTTTCGAAAAGGAAAGATTGTTCAAAACATCACATAGTATGGGAATGATAGGGAAAAGGCATGCTCTTAAAACAAGTCCAAAAATAGATAAAGTCGTAGTAGATCTTGAAACACTGTTT
Allele-29	GGTTTCGACAGGGATGCATTGTTCAAATCATCACATGGAATGGGAAAGGTAGGTAAAATGCATGGTCTTAAAACGACTCCAAAAGTAGAGAGAGTCTTAAAAGATCTTGAAACACTGTTT
Allele-30	GGTTTCGATAGGCAAAGATTGTTCTTAACATCTAAGAGTATGAGTGAAGTAGGGAGAAGGCATGGTATTAGACCTGGAACAAAAACAGAAAAATATCTAAAAGAACTTGCTAGTCTTCTT
Allele-31	AATTTAGATAAGGATAAATTGTTCATGACATCTAAGGCTATGACGGCGACAGGGAGAAGGCATGGTATTAGACCTGGAACTTCAAAAGAGAAATTTACTAGTGAACTTGCTAAACTTCTT
Allele-32	CATCCTGACAAGGAAAGATTGTTCAAGACATCAAAGGGTATGACAGAAGTAGGTAAAAAGCATGGCATTAAAATGGGAACATCGATAGATAAATTCCTAAGTGAACTTCAAAAATTATTT
Allele-33	GGTTTCGACAGGGATGCATTGTTCAAATCATCACATGGTATGGGAAAGGTAGGAAAAAGGTATGGTCTTAAAACTACTCCAAAAGTAGATAAAGTCTTAGCAGATCTTGAAACACTGTTT
Allele-34	GGTTTCGATAGGGAGCTATTGTTCAAATCATCACATGGAATGGGAAAGATAGGTAGAAAGCATGGTCTTAAAACAAAACCAAGATTAGAGAGTGTATTAGAAGATCTTGAAAAACTGTTT
Allele-35	GATTTTGAAAAGGAAAAATTGTTCGCGGCATCACATGGATTGGGAAAGGTAGGGAAAACTTTGGTTTAAAGATAAAACCAAAAGTAGATCATATCTTAAATGATATTATGAAAGTGTTT
Allele-36	GGTTTCGACAGGGATGCATTGTTCAAATCATCACATGGTATGGGAAAGGTAGGAAAAAGGTATGGTCTTAAAACTACTCCAAAAGTAGATAAAGTCTTAGCAGATCTTGAAACACTGTTT
Allele-37	GATCCTGACAAGGAAACATTGTTCAAAACATCAAAGGCTATGACGGCGACAGGGAGAAAGCATGGTATTAGACCTGGAACAACAAAAGAGAAATTTACTAGTGAACTTGCTAAACTTCTT
Allele-38	GGTTTCGACAGGGACGCATTGTTCAAATCATCACATGGTATGGGAAAGGTAGGAAAAAGGTATGGTCTTAAAACTACTCCAAAAGTAGATAAAGTCTTAGCAGATCTTGAAACACTGTTT
Allele-39	GATCCTAACAAGGAAAGATTGTTCTTAACATCTAAGTCTATGATGACAGTAGGGAAAAGGTATGGTCTTCGACTTGGAACATCAAAAGAGAAATTTCTAAAAGAGCTTAATAGTTTTCTT
Allele-40	GGTTTCGATAAGGAACTATTGTTTAAATCATCAAAGGGTATGACAGCGGTAGGGAAAAAGTATGGTATTAGGCCAGGAACATCGGTAGATAAATTTCAAAGAGAACTTCAAATATTATTT
Allele-41	GGTTTCGAAAAGGAAAGATTGTTCAAAACATCACATAGTATGGGAATGATAGGGAAAAGGCATGCTCTTAAAGCAAGTCCAAAAATAGATAAAGTCGTAGTAGATCTTGAAACCCTGTTT
Allele-42	GGTTTCGAAAAGGAAAGATTGTTCAAAACATCACATAGTATGGGAATGATAGGGAAAAGACATGATCATAAAGCAAGTCCAAAAATAGATAAAGTCGTAGTAGATCTTGAAACACTGTTT
Allele-43	GGTTTAGATATGGAGGCATTGTTCAAAACATCTAAGGGTATGACGAAAGTAGGGAGAAAGTATGGTATTAGACCTGGAACTACAAAAGATAAACTTACTAGAGAACTTACTAGACTTCTT
	* * * ****** **** * ** *** * ** ** ** ** * * **

```
                   241                                                                                                                   360
Allele-1[24]       GGAAAACACGGTCTTGGTGGTATTAGTAAAGATTGTCTTAAATGTTTTGCACAAAGCCTAGTGTGCGTATTAATGAAATGTAGAGGAGCATGTCTCAAAGGACCATGTACTGACGACTGC
Allele-2[8]        ACAGAAGTCGGTATTACAGGTGTTGGTGAGAAGTGCCTCGAATGTTTCGCAGCAAGTATTAAGTGTGTAGCACAATATTGCAAAGGAGCGTGTCTAAAGGGACCATGTACAGAAGACTGC
Allele-3[4]        GGAAAACACGGTCTTGGTGGTATTAGTAAAGATTGTCTTAAATGTTTTGCACAAAGCCTAGTGTGCGTATTAATGAAATGTAGAGGAGCATGTCTCAAAGGACCATGTACTGACGACTGC
Allele-4[2]        ACAGAAATTGGTATTACAGGTGTTGGTGAGAAGTGCCTCGAATGTTTAGCAGCAAGCATTAAGTGTGTATCACACCATTGCAAGGGAGCGTGTCTAAAGGGACCATGTACAGAAGGCTGT
Allele-5           GGAAAACACGGTCTTGGTGGTATTAGTAAAGATTGTCTTAAATGTTTTGCACAAAGCCTAGTGTGCGTATTAATGAAATGTAGAGGAGCATGTCTCAAAGGACCATGTACTGACGACTGC
Allele-6[3]        GAAAAACACGGTCTTGGTGGTGTTAGTAAAGATTGTCTTAAATGTTTTGGACAAAGCGTAGTGTGCGTATTAATGAGATGTAGAGGAGCATGTCTCAAAGGACCATGTTCTAAAGACTGT
Allele-7[2]        GGAAGCCTGGGTATTTCAGGAGTTAGTGAACAATGTCTTGGCTGTTTTGCACAAAGCATTAAGTGTGTATCACAACATTGCAAGGGAGCGTGTCTTAGGGGTCCATGTACTCAAGACTGC
Allele-8           GGAAAAATCGGTCTTGGTGGTATTAGTAAAGATTGTCTTAAATGTTTTGTGGAAAGCATTTTGTGTGTTATAAAAAAATGTAGAACAGCATGTCTCAGAGGACCATGTACTGATGACTGC
Allele-9           GGAAAAATCGGTCTTGTTGGTGTTAGTAAAACTTGTCTTAAATGTTTTGCACAAAGCATTAAGTGTGTAGCACAACATTGCAAAGGAGCGTGTCTAAAGGGACCATGTACAGAAGACTGC
Allele-10          GAAAAACACGGTCTTGGTGGTGTTAGTAAAGATTGTCTTAAATGTTTTGGACAAAGCGTAGTGTGCGTATTAATGAGATGTAGAGGAGCATGTCTCAAAGGACCATGTTCTAAAGACTGT
Allele-11          GGAAAACACGGTCTTGGTGGTATTAGTAAAGATTGTCTTAAATGTTTTGCACAAAGCCTAGTGTGCGTATTAATGAAATGTAGAGGAGCACGTCTCAAGGGACCATGTACTGACGACTGC
Allele-12          GAAAAAATCGGTATTACAGGAGTTAGTGAGTCTTGTCTTAGTTGTTTTGGACAAAGCATTAAGTGCGTAGCACAAAAATGCAAAGGAGAGTGTCTTAGGGGTCCATGTACACAAGACTGC
Allele-13          AGAACCATCGGCATTACAGGAGTTAGTGAGCCTTGTCTTCTATGTTTTGGACAAAGCATTAAGTGTGTAGTACAAAAATGCAAAGGAGAGTGCCTTAGGGGTCCATGTAGTCAAGGCTGT
Allele-14          GGAAAACACGGTCTTGGTGGTATTAGTAAAGATTGTCTTAAATGTTTTGCACAAAGCCTAGTGTGCGTATTAATGAAATGTAGAGGAGCATGTCTCAAAGGACCATGTACTGACGACTGC
Allele-15          GGAAAAATCGGTCTTGGTGGTATTAGTAAAGATTGTCTTAAATGTTTTGTGGAAAGCATTTTGTGTGTTATAAAAAAATGTAGAACAGCATGTCTCAAAGGACCATGTACTGATGACTGC
Allele-16          ACACAACTAGGTATTACAGGAATTAGAGAACAATGTCTTGCCTGTTATGCACAAAGCATATATTGCGTTGCGAATAATTGCAGGGGAGCATGTCTTAGGGGTCCATGTACTAAAGAGTGT
Allele-17          GGAAAACACGGTCTTGGTGGTATTAGTAAAGATTGTCTTAAATTGTTTGCACAAAGCCTAGTGTGCGTATTAATGAATTGTAGAGGAGCATGTCTCAAAGGACCATGTACTGACGACTGC
Allele-18          AGAAGCATCGGTATTGTAGAAGTTAGTGATAAGTGCCTCGAATGTTTTGGAGCAAGCATCCAGTGCGTTGTAAAAAAATTGCAGGGGAGATTGTATTAAGGGTCCATGTACTTCGGGCTGC
Allele-19          GCCTCCTGTGGAATTACAGGAATTAGAGAACAATGTCTTGCTTGTTTTGCACAAAGCATATATTGCGTTGCAAATAATTGCAGGGGAGCATGTCTTAGGGGTCCATGTACTAAAGAGTGT
Allele-20          GGAAAACACGGTCTTGGTGGTATTAGTAAAGATTGTCTTAAATGTTTTGCACAAAGCCTAGTGTGCGTATTAATGAAATGTAGAGGAGCATGTCTCAAAGGACCATGTACTGACGACTGC
Allele-21          AGAAGCATCGGTATTGTAGAAGTTAGTGATAAGTGCCTCGAATGTTTTGGAGCAAGCATCCAGTGCGTTGTAAAAAAATTGCAGGGGAGATTGTATTAAGGGTCCATGTACTTCGGGCTGC
Allele-22          GAAAAACTCGGTCTTGTAGGAATTAGTGAGTCTTGTCTTAGTTGTTTTGGACAAAGCCTTAAGTGTGTATTACAGCATTGCAAGGGAGCGTGTCTTAGGGGACCATGTACTGAAGACTGC
Allele-23          GGAAAAATCGGTCTTGGTGGTATTAGTAAAGATTGTCTTAAATGTTTTGTGGAAAGCATTATGTGTGTTATAAAAAAATGTAGAACAGCATGTCTCAGAGGACCATGTACTGATGACTGC
Allele-24          AGAACCATCGGCATTACAGGAGTTAGTGAGCCTTGTCTTCTATGTTTTGGACAAAGCATTAAGTGTGTAGTACAAAAATGCAAAGGAGAGTGCCTTAGGGGTCCATGTAGTCAAGGCTGT
Allele-25          GGAAAACACGGTCTTGGTGGTATTAGTAAAGATTGTCTTAAATGTTTTGCACAAAGCCTAGTGTGCGTATTAATGAAATGTAGAGGAGCATGTCTCAAAGGACCATGTACTGACGACTGC
Allele-26          GAAAAAATCGGTATTATAGGAGTTAGTGAGTCTTGTCTTAGTTGTTTTGGACAAAGCATTAAGTGCGTAGCACAACATTGCAAGGGAGCGTGTCTTAGGGGACCATGTACAGAAGACTGC
Allele-27          GGACAAATTGGTATTTCAGGAGTTAGTACACAATGTCTTCGATGTTTTGCACAGAGCATATATTGCGTTGTGAAAAATTGCAGGGGATCATGTATGAAGGGTCCATGCAAACCAGAATGC
Allele-28          GAAAAACACGGTCTTGGTGGTGTTAGTAAAGATTGTCTTAAATGTTTTGGACAAAGCGTAGTGTGCGTATTAATGAGATGTAGAGGAGCATGTCTCAAAGGACCATGTTCTAAAGACTGT
Allele-29          GGAAAAATCGGTCTTGGTGGTATTAGTAAAGATTGTCTTAAATGTTTTGTGGAAAGCATTATGTGTGTTATAAAAAAATGTAAAACAGCATGTCTCAAAGGACCATGTACTGATGACTGC
Allele-30          ACAAAACTAGGTATTACAGGAATTAGTGAAGGTTGTCTCGGTTGTTTTGCACAAAGCATATATTGCGTTGTGAAAAATTGCAAAGGAGCGTGTCTTAAGGGTCCATGTAAACCAGACTGC
Allele-31          GGAAGCATCGGCATTACAGGAGTTAGTGAGCCTTGTCTTGGTTGTTTTGGACAAAGCATTAAGTGTGTAGTACAAAAATGCAAAGGAGATTGCCTTAAGGGTCCATGCACTTCGGGCTGC
Allele-32          GGACAAGTTGGTATTTCAGGAGTTGGTGAAGAATGTCTTCGATGTTTTGCAGCAAGCATCCATTGTGTTTCGAACAAATGTAAAGGAGCGTGTCTTAGGGGTCCATGTACAGATGACTGT
Allele-33          GGAAAACACGGTCTTGGTGGTATTAGTAAAGATTGTCTTAAATGTTTTGCACAAAGCCTAGTGTGCGTATTAATGAAATGTAGAGGAGCATGTCTCAAAGGACCATGTACTGACGACTGC
Allele-34          GGACAACACGGTCTTGGTGATATTAGTAAAAATTGTCTTAAATGTCTTGCACAAAGCATAGTGTGCGTTGTAAGTAAATGTAGAGCAGCATGTCTCAAAGGACCATGTACAGACGGCTGC
Allele-35          GGAGAATACGGTATTGAAGGGGTGAGTGCAAAGTGCCTTCCATGTTTTGCACAAAGCCTCCTATGTTTTCTACAAAATTGTAGGGGACCATGTCTCGGAGACCCATGTAGTGAAGGATGT
Allele-36          GGAAAACACGGTCTTGGTGGTATTAGTAAAGATTGTCTTAAATGTTTTGCACAAAGCCTAGTGTGCGTATTAATGAAATGTAGAGGAGCATGTCTCAAAGGACCATGTACTGACGACTGC
Allele-37          GGAAGCATCGGCATTACAGGAGTTGGTGAGGCTTGTCTTGGTTGTTTTGGGCAAAGCATTAAGTGTGTAGTACAAAAATGCAAAGGAGATTGCCTTAAGGGTCCATGCACTTCGGGCTGC
Allele-38          GGAAAACACGGTCTTGGTGGTATTAGTAAAGATTGTCTTAAATGTTTTGCACAAAGCCTAGTGTGCGTATTAATGAAATGTAGAGGAGCATGTCTCAAAGGACCATGTACTGACGACTGC
Allele-39          ACAAAACTAGGTATTACAGGAATTAGTGAAGAATGTCTCGGTTGTTTTGCACAAAGTATTAAGTGTGTATCACAACATTGCAAGGGAGCGTGTCTAAAGGGACCATGTACAGATGACTGC
Allele-40          CGACAAATTGGTATTTCAGAAGTTAGTGCAGAATGTCTTAAATGTTTTGCAAAAAGCATTAAGTGTGTATCACAACATTGCAAAGGAGAGTGCCTTAAGGGTCCATGTAGTAAAGGCTGT
Allele-41          GAAAAACACGGTCTTGGTGGTGTTAGTAAAGATTGTCTTAAATGTTTTGGACAAAGCGTAGTGTGCGTATTAATGAGATGTAGAGGACCCTGTCTCAAAGGACCATGTTCTAAAGACTGT
Allele-42          GAAAAACACGGTCTTGGTGGTGTTAGTAAAGATTGTCTTAAATGTTTTGGACAAAGCGTAGTGTGCGTATTAATGAGATGTAGAGGAGCATGTCTCAAAGGACCATGTTCTAAAGACTGT
Allele-43          GAAAAAATCGGTATTATAGGAGTTAGTGAGTCTTGTCTTAGTTGTTTTGGACAAAGCATTAAGTGTGTAGCACAACATTGCAAGGGAGCGTGTCTTAGGGGACCATGTACAGAAGACTGC
                            **  **   *   *  *       ** **    *     *     **  *    **  *          ** *    *    *  *    *  *****       *  **
```

```
                  361                                                                                                                      480
Allele-1[24]      CAAAATTGCTTTGATAGAAACTGTAAATCTGCATTGCTGGAATGCATTGGGAAAACAAGTATTCCAAATCCATGTAAATGGAAAGAAGATTATCTAAAATACAAATTTCCTGAAACAGAT
Allele-2[8]       CAACAATGCATTAAATCTAATTGTATGGACGGATTGCTGGAATGTATTGGTAAACCCAGTGTTCCAAACCCTTGTGATTGGAAAGATGCTTATCTAAAATTCAAACTTCCTGAAACAGGT
Allele-3[4]       CAAAATTGCTTTGATAGAAACTGTAAATCTGCATTGCTGGAATGCATTGGGAAAACAAGTATTCCAAATCCATGTAAATGGAAAGAAGATTATCTAAAATACAAATTTCCTGAAACAGAT
Allele-4[2]       CAAGAATGCATTAAAAGAAATTGTATGGAAGCATTGCTGCAATGCATTGGGAAACCAAGTGTTCCAAATCCTTGTGATTGGAAAGATGATTATCTAAAATTCAAATTTCCTGAAACAGGT
Allele-5          CAAAATTGCTTTGATAGAAACTGTAAATCTGCATTGCTGGAATGCATTGGGAAAACAAGTATTCCAAATCCATGTAAATGGAAAGAAGATTATCTAAAATACAAATTTCCTGAAACAGAT
Allele-6[3]       CAAGAATGCATTAAAAGAAATTGTAGGCAAGGATTGCTGGAATGCATTGGAAAAGAGGATGTTCCAAATCCTTGCGATTGGGACAAAGAATATCTAAAATACAGACTTCCTGATACAGAT
Allele-7[2]       CAAAAATGCATTGAAAGAAATTGTAAAGATGCATTACTGGAATGTATTGGGAAACAGAGTATTCTGAATCCTTGTACATGGGAAAGTGAATATCTAAAATTCAAACTTCCTGAAACAGGT
Allele-8          CAAAATTGTATTAAAAAAAATTGTAAGCAAGGATTGCTGGAATGCATTGGAAAAGAAGATGTTCCAAATCCTTGCGATTGGGACAAAGAATATCTAAAATACAAACTTCCTGATACAGAT
Allele-9          CAAAAATGCATTAATAAAAATTGTAAGCAGGCATTGCTGGAATGCATTGGGAAAACAAATATTCCAAACCCTTGTGATTGGAACAAAGCATATCTAAAATTCAAACTTCCTGAAACAGGT
Allele-10         CAAGAATGCATTAAAAGAAATTGTAGGCAAGGATTGCTGGAATGCATTGGAAAAGAGGATGTTCCAAATCCTTGCGATTGGGACAAAGAATATCTAAAATACAGACTTCCTGATACAGAT
Allele-11         CAAAATTGCTTTGATAGAAACTGTAAATCTGCATTGCTGGAATGCATTGGGAAAACAAGTATTCCAAATCCATGTAAATGGAAAGAAGATTATCTAAAATACAAATTTCCTGAAACAGAT
Allele-12         CAAAAATGCATTGAAAAAAATTGTAAACCGGCATTGCTGGAATGTATTGGAAAAAGTAATATTCCGAATCCTTGTAAATGGGAAAAAGAATATCTAAATTACAAACTTCCTGAAACAGAT
Allele-13         CAAAAGTGTATTGATGCTAATTGTAAACCGGCATTGGTGGAGTGTGTTGGAAATAATAACATTCTGAATCCCTGTAAATGGGAAGATAAATATCTAAAATTCAAAATTCCTGGAACAGGT
Allele-14         CAAAATTGCTTTGATAGAAACTGTAAATCTGCATTGCTGGAATGCATTGGGAAAACAAGTATTCCAAATCCATGTAAATGGAAAGAAGATTATCTAAAATACAAATTTCCTGAAACAGAT
Allele-15         CAAAATTGTATTAAAAAAAATTGTAAGCAAGGATTGCTGGAATGCATTGGAAAAGAAGATGTTCCAAATCCTTGCGATTGGGACAAAGAATATCTAAAATACAAACTTCCTGATACAGAT
Allele-16         CAAGAATGCATTAAAAAAAATTGTAAACAGGCATTGCTGGAATGTATTGGAAAAGGTGATGTTCCGAATCCTTGTCAATGGAAAGATGATTATCTAAAATTCAAACTTCCTGAAACAGAT
Allele-17         CAAAATTGCTTTGATAGAAACTGTAAATCTGCATTGCTGGAATGCATTGGGAAACAAAGTATTCTAAATCCTTGTACATGGAAAGAAGATTATCTAAAATACAAATTTCCTGAAACAGAT
Allele-18         CAAAATTGCGTTAAAACTAAATGTAAGCCAAAAATGCTGGAATGTATTGGAACAAGTGATGTTCCAAATCCTTGTGAATGGGAATGTGAATATCTAAAATACAAACTTCCTGAAACAGGT
Allele-19         CAAGAATGCATTAAAAAAAATTGTAAACAGGCATTGCTGGAATGTATTGGAAAAGGTGATGTTCCGAATCCTTGTCAATGGATATATGATTATCTAAAATTCAAACTTCCTGAAACAGAT
Allele-20         CAAAATTGCTTTGATAGAAACTGTAAATCTGCATTGCTGGAATGCATTGGGAAAACAAGTATTCCAAATCCATGTAAATGGAAAGAAGATTATCTAAAATACAAATTTCCTGAAACAGAT
Allele-21         CAAAATTGCGTTAAAACTAAATGTAAGCCAAAAATGCTGGAATGTATTGGAACAAGTGATGTTCCAAATCCTTGTGAATGGGAAGGTGAATATCTAAAATACAAACTTCCTGAAACAGGT
Allele-22         CAAAAATGCTTTGAAAAAAATTGTAAACCTGCATTGCTGGAATGCATTGGAAAAGGTGATGTTCCAAATCCATGCGATTGGAAGGAAGATTATCTAAGATACAAACTTCCTGAAACAGAT
Allele-23         CAAAATTGCATTAAAAGAAATTGTAAGCAAGGATTGCTGGAATGCATTGGAAAAGAGGATGTTCCAAATCCTTGCGATTGGGACAAAGAATATCTAAAATACAAACTTCCTGATACAGAT
Allele-24         CAAAAGTGTATTGATGCTAATTGTAAACCGGCATTGGTGGAGTGTGTTGGAAATAATAACATTCTGAATCCCTGTAAATGGGAAGATGAATATCTAAAATTCAAAATTCCTGGAACAGGT
Allele-25         CAAAATTGCTTTGATAGAAACTGTAAATCTGCATTGCTGGAATGCATTGGGAAAACAAGTATTCCAAATCCATGTAAATGGAAAGAAGATTATCTAAAATACAAATTTCCTGAAACAGAT
Allele-26         CAAAAATGCATTGAAAAAAATTGTAAACAGGCATTGCTGGAATGTATTGGAAAAGGTGATGTTCCGAATCCTTGCGATTGGGAGAAAGAATATCTAAGTTACAAACTTCCTGAAACAGAT
Allele-27         CAAAATTGCATTGAAAAAAATTGTAAAAAGGCATTCTTGGAATGTATTGGGAAAGATAATACACCTAATCCCTGTAATTGGGAAAGTGAATATCTAAAATACAAACTTCCTGAAACAGGT
Allele-28         CAAGAATGCATTAAAAGAAATTGTAGGCAAGGATTGCTGGAATGCATTGGAAAAGAGGATGTTCCAAATCCTTGCGATTGGGACAAAGAATATCTAAAATACAGACTTCCTGATACAGAT
Allele-29         CAAAATTGCATTAAAAGAAATTGTAAGCAAGGATTGCTGGAATGCATTGGAAAAGAGGATGTTCCAAATCCTTGCGATTGGGACAAAGAATATCTAAAATACAAACTTCCTGATACAGAT
Allele-30         CAAAGTTGCATTACAAAAAATTGTAAAACGAAATTCCTGGAATGTATTGGGAAAGGTGATGCCCCTAATCCTTGTACGTGGGAAGCTGATTATCTAAAATACAAACTTCCTGAAACAGAT
Allele-31         CAAAATTGCGTTAAAACTAAATGTAAACCGGCATTGCTGGAATGCATTGGAAAAGAAGATGTTCCAAATCCTTGCGAATGGGAAAGTGAATATCTAAAATACAAACTTCCTGAAACAGGT
Allele-32         CAAAAATGCATTGAAAGAAATTGTAAAGGGGCATTGCTGGAATGTATTGGTAAACCGAAGATTCCAAACCCTTGTGATTGGAAAGATGAGTATCTAAAATTCAAATTTCCTACAACAGAT
Allele-33         CAAAATTGCTTTGATAGAAACTGTAAATCTGCATTGCTGGAATGCATTGGGAAAACAAGTATTCCAAATCCATGTAAATGGAAAGAAGATTATCTAAAATACAAATTTCCTGAAACAGAT
Allele-34         CAAAGATGTATTGAAGCTAATTGTAAAAAGGCATTGCTGGAGTGTATTGGAACAAGTGATATTCCAAATCCTTGTAAATGGGAAAGTGATTATCTAAAATACAAACTTCCTGATACAGAT
Allele-35         CAAAATTGTTTTAAAAGAAACTGTGAGGAAGGATTGCTGAAGTGTATTGGGAAAGACAAAATTAAGAACCCTTGTGATTATGGAGGTGAGTATTTAAAATACAAACCGCAGAAAACAGAT
Allele-36         CAAAATTGCTTTGATAGAAACTGTAAATCTGCATTGCTGGAATGCATTGGGAAAACAAGTATTCCAAATCCATGTAAATGGAAAGAAGATTATCTAAAATACAAATTTCCTGAAACAGAT
Allele-37         CAAAATTGCGTTAAAACTAAATGTAAACCGGCATTGCTGGAATGCATTGGAAAAGAAGATGTTCCAAATCCTTGCGAATGGGAAAGTGAATATCTAAAATACAAACTTCCTGAAACAGGT
Allele-38         CAAAATTGCTTTGATAGAAACTGTAAATCTGCATTGCTGGAATGCATTGGGAAAACAAGTATTCCAAATCCATGTAAATGGAAAGAAGATTATCTAAAATACAAATTTCCTGAAACAGAT
Allele-39         CAACAATGCATTAAAAGAAATTGTATGGACGCACTGCTGGAATGTGTTGGGAAACAGAGTATTCTGAATCCTTGTAATTGGGAAAATGAATATCTAAAATACAAACTTCCTGAAACAGGT
Allele-40         CAAAATTGTATTGATACACATTGTAAACCGGCATTGCTGGAGTGTATTGGGAAAACAAGTATTCCAAATCCATGTAAATGGAAAGAAGATTATCTAAAATACAAATTTCCTGAAACAGAT
Allele-41         CAAGAATGCATTAAAAGAAATTGTAGGCAAGGATTGCTGGAACGCATTGGAAAAGAGGATGTTCCAAATCCATGTAAATGGAAAGAAGATTATCTAAAATACAAATTTCCTGAAACAGAT
Allele-42         CAAGAATGCATTAAAAGAAATTGTAGGCAAGGATTGCTGGAACGCATTGGAAAAGAGGATGTTCCAAATCCTTGCGATTGGGACAAAGAATATCTAAAATACAGACTTCCTGATACAGAT
Allele-43         CAAAAATGCATTGAAAAAAATTGTAAACAGGCATTGCTGGAATGTATTGGAAAAGGTGATGTTCCGAATCCTTGCGATTGGGAGAAAGAATATCTAAAATACAAATTTCCTGAAACAGAT
                  ***   **  **       * ***        * *  ** *  *  **** *              ** ** **    *           *** ***  * ** *   *     **** *
```

```
                  481                                          525
Allele-1[24]        GAGGACGAATCTACGAAAAAAAGGAGAAGCCTCCGGCACTTCATAG
Allele-2[8]         GAGGGCGAATCTGAGAAAAAAAGGAGAAGCCTCCGGCACTTCATAG
Allele-3[4]         GAGGACGAATCTACGAAAAAAAGGAGAAGCCTCCGGCACTTCATAG
Allele-4[2]         GAGGATGAGGCTCAGAAAAAAAGGAGAAGCCTCCGGCACTTCATAG
Allele-5            GAGGACGAATCTCAGAAAAAAAGGAGAAGCCTCCGGCACTTCATAG
Allele-6[3]         GAGGATGAGTCTCAGAAAAAAAGGAGAAGCCTCCGGCACTTCATAG
Allele-7[2]         GAGGACGAATCTGAGAAAAAAAGGAGAAGCCTCCGGCACTTCATAG
Allele-8            GGGGATGAGTCTCAGAAAAAAAGGAGAAGCCTCCGGCACTTCATAG
Allele-9            GAGGACGAATCTCAGAAAAAAAGGAGAAGCCTCCGGCACTTCATAG
Allele-10           GAGGATGAGTCTCAGAAAAAAAGGAGAAGCCTCCGGCACTTCATAG
Allele-11           GAGGACGAATCTACGAAAAAAAGGAGAAGCCTCCGGCACTTCATAG
Allele-12           GAGGACGAATCTGAGAAAAAAAGGAGAAGCCTCCGGCACTTCATAG
Allele-13           GAGGACGAATCTGAGAAAAAAAGGAGAAGCCTCCGGCACTTCATAG
Allele-14           GAGGACGAATCTGAGAAAAAAAGGAGAAGCCTCCGGCACTTCATAG
Allele-15           GGGGATGAGTCTCAGAAAAAAAGGAGAAGCCTCCGGCACTTCATAG
Allele-16           GAGGAAGAATCTGAGAAAAAAAGGAGAAGCCTCCGGCACTTCATAG
Allele-17           GAGGACGAATCTGAGAAAAAAAGGAGAAGCCTCCGGCACTTCATAG
Allele-18           GAGGACGAATCTGAGAAAAAAAGGAGAAGCCTCCGGCACTTCATAG
Allele-19           GAGGAAGAATCTGAGAAAAAAAGGAGAAGCCTCCGGCACTTCATAG
Allele-20           GAGGACGAATCTACGAAAAAAAGGAGAAGCCTCCGGCACTTCATAG
Allele-21           GAGGACGAATCTGAGAAAAAAAGGAGAAGCCTCCGGCACTTCATAG
Allele-22           GAGGACGAATCTGAGAAAAAAAGGAGAAGCCTCCGGCACTTCATAG
Allele-23           GGGGATGAGTCTCAGAAAAAAAGGAGAAGCCTCCGGCACTTCATAG
Allele-24           GAGGACGAATCTGAGAAAAAAAGGAGAAGCCTCCGGCACTTCATAG
Allele-25           GAGGACGAATCTGAGAAAAAAAGGAGAAGCCTCCGGCACTTCATAG
Allele-26           GAGGACGAATCTGAGAAAAAAAGGAGAAGCCTCCGGCACTTCATAG
Allele-27           GAGGACGAATCTGAGAAAAAAAGGAGAAGCCTCCGGCACTTCATAG
Allele-28           GAGGATGAGTCTCAGAAAAAAAGGAGAAGCCTCCGGCACTTCATAG
Allele-29           GGGGATGAGTCTCAGAAAAAAAGGAGAAGCCTCCGGCACTTCATAG
Allele-30           GAGGACGAATCTGAGAAAAAAAGGAGAAGCCTCCGGCACTTCATAG
Allele-31           GAGGAAGAATCTGAGAAAAAAAGGAGAAGCCTCCGGCACTTCATAG
Allele-32           AGTGACAAATCTGAGAAAAAAAGGAGAAGCCTCCGGCACTTCATAG
Allele-33           GAGGACGAATCTACGAAAAAAAGGAGAAGCCTCCGGCACTTCATAG
Allele-34           GAGGACGAATCTGAGAAAAAAAGGAGAAGCCTCCGGCACTTCATAG
Allele-35           GGGGACGAATCTGCGAAAAAAAGGAGAAGCCTCCGGCACTTCATAG
Allele-36           GAGGACGAATCTGAGAAAAAAAGGAGAAGCCTCCGGCACTTCATAG
Allele-37           GAGGAAGAATCTGAGAAAAAAAGGAGAAGCCTCCGGCACTTCATAG
Allele-38           GAGGACGAATCTACGAAAAAAAGGAGAAGCCTCCGGCACTTCATAG
Allele-39           GAGGAAGAATCTGAGAAAAAAAGGAGAAGCCTCCGGCACTTCATAG
Allele-40           GAGGACGAATCTACGAAAAAAAGGAGAAGCCTCCGGCACTTCATAG
Allele-41           GAGGACGAATCTACGAAAAAAAGGAGAAGCCTCCGGCACTTCATAG
Allele-42           GAGGACGAATCTACGAAAAAAAGGAGAAGCCTCCGGCACTTCATAG
Allele-43           GAGGACGAATCTGAGAAAAAAAGGAGAAGCCTCCGGCACTTCATAG
                       *   *  **  *******************************
```

Figure S2. Multiple sequence alignment of 43 Tp2 alleles obtained in this study
