# Supplementary material for: Two Theileria parva CD8 T Cell Antigen Genes Are More Variable in Buffalo than Cattle Parasites, but Differ in Pattern of Sequence Diversity
Source: PLoS One. 2011 Apr 29;6(4):e19015. doi: 10.1371/journal.pone.0019015 (PMC3084734; doi:10.1371/journal.pone.0019015)
Supplement: Table S1 — Cell lines infected with T. parva cattle-derived parasite stocks isolated from different geographic areas of eastern and southern Africa. A. Laboratory samples. B. Kenyan field isolates from cattle with no association with buffalo. C. Isolates derived from buffalo. (DOC) [file pone.0019015.s003.doc]

**Table S1.** Cell lines infected with *T*. *parva* cattle-derived parasite stocks isolated from different geographic areas of eastern and southern Africa.

**A**  Laboratory samples.

| **Sample** | **Origin** | **Stabilate (year isolated)** | **Cell line (year isolated)** | **Tp1 alleles** | | | | **Tp2 alleles** | | |
| --- | --- | --- | --- | --- | --- | --- | --- | --- | --- | --- |
|  |  |  |  | **gene** | **GenBank Acc. No.**^$^ | **antigen** | **CTL epitope^γ^** | **gene** | **GenBank Acc. No.** | **antigen** |
| LS1 | Muguga | 3087 (1961) | F100 (1987) | 1 | JF451936 | 1 | ML | 1 | JF451856 | 1 |
| LS2 | Marikebuni | 4108 (1981) | 1677 (2002) | 2 | JF451960 | 2 | **II** | 1 | JF451857 | 1 |
| LS3 | Marikebuni | 2245 (1981) | C887 (1985) | 2 | JF451961 | 2 | **II** | 1 | JF451858 | 1 |
| LS4 | Marikebuni | 3292 (1981) | BJ253 (1992) ^ß^ | 2 | JF451962 | 2 | **II** | 1 | JF451859 | 1 |
| LS5 | Mariakani | 3319 (1981) | B506 (1982) | 1 | JF451937 | 1 | ML | 1 | JF451860 | 1 |
| LS6 | Muguga | 73 (1961) | BN64 (1996) ^ß^ | 1 | JF451938 | 1 | ML | 1 | JF451861 | 1 |
| LS7 | Kiambu 5 | 68 (1971) | BN65 (1996) ^ß^ | 1 | JF451939 | 1 | ML | 2 | JF451880 | 2 |
| LS8 | Serengeti (Ta) ^α^ | 69 (1972) | BN140 (1996) ^ß^ | 1 | JF451940 | 1 | ML | 1 | JF451862 | 1 |
| LS9 | Chitongo (Za) | Zambia 2 (1982) | Pal 213 Za 2 (1982) ^ß^ | 5 | JF451975 | 10 | **II** | 3 | JF451884 | 3 |
| LS10 | Katete (Za) | V8 (1982) | F280 Katete (1989) ^ß^ | nd | na | nd | na | 1 | JF451863 | 1 |
| LS11 | Boleni (Zi) | 2628 (1984) | D602 Bol (1985) ^ß^ | 2 | JF451963 | 2 | **II** | 3 | JF451885 | 3 |
| LS12 | Uganda (Ug) | 3645 (1976) | BH 312 (1992) ^ß^ | 3 | JF451971 | 2 | **II** | 3 | JF451886 | 3 |
| LS13 | Boleni (Zi) | 3039 (1984) | D409 Boleni (1992) | 2 | JF451964 | 2 | **II** | 3 | JF451887 | 3 |
| LS14 | Mariakani | 3319 (1982) | D409 TpB (1987) | 2 | JF451965 | 2 | **II** | 3 | JF451888 | 3 |
| LS15 | Marikebuni | 3014 (1981) | D409 Tp214 (1987) | 2 | JF451966 | 2 | **II** | 1 | JF451864 | 1 |
| LS16 | Uganda (Ug) | 3066 (1976) | D409 TpD (1987) | 2 | JF451967 | 2 | **II** | 3 | JF451889 | 3 |
| LS17 | Nanyuki | 3081 (1972) | D409 TpE (1987) | 1 | JF451941 | 1 | ML | 5 | JF451894 | 5 |

^α^ Serengeti; Serengeti-transformed. Cell lines not established by *in vitro* infection but from biopsies are indicated with ^ß^.

^γ^ Polymorphic amino acid residues present in the Tp1 CTL epitope.

Samples were from Kenya or otherwise indicated (Ta: Tanzania; Ug: Uganda; Za: Zambia; Zi: Zimbabwe).

^$^GenBank Acc. No.; Genbank accession numbers.

nd, not done; na, not applicable.

**B** Kenyan field isolates from cattle with no association with buffalo.

| **Sample** | **Origin** | **Animal** | **Year**  **isolated** | **Tp1 alleles** | | | | **Tp2 alleles** | | |
| --- | --- | --- | --- | --- | --- | --- | --- | --- | --- | --- |
|  |  |  |  | **gene** | **GenBank Acc. No.** | **antigen** | **CTL epitope** | **gene** | **GenBank Acc. No.** | **antigen** |
| CD1 | Kakuzi^a^ | BR 521 | 1998 | 1 | JF451942 | 1 | ML | 3 | JF451890 | 3 |
| CD2 | Kakuzi | BR 498 | 1998 | nd |  | nd | na | 3 | JF451891 | 3 |
| CD3 | Kakuzi | BR 476 | 1998 | 1 | JF451943 | 1 | ML | 1 | JF451865 | 1 |
| CD4 | Kakuzi | BR 238 B4^δ^ | 1998 | 1 | JF451944 | 1 | ML | 1 | JF451866 | 1 |
| CD5 | Kakuzi | BR238 G12^δ^ | 1998 | 1 | JF451945 | 1 | ML | nd | na | nd |
| CD6 | Kilifi^a^ | KL 2 | 1997 | 4 | JF451973 | 3 | II | 1 | JF451867 | 1 |
| CD7 | Kilifi | BR 293 | 1997 | 1 | JF451946 | 1 | ML | 1 | JF451868 | 1 |
| CD8 | Kilifi | KL 5 | 1997 | 1 | JF451947 | 1 | ML | 1 | JF451869 | 1 |
| CD9 | Kilifi | BR 114 | 1997 | 1 | JF451948 | 1 | ML | 1 | JF451870 | 1 |
| CD10 | Kilifi | BR 125 | 1997 | 1 | JF451949 | 1 | ML | nd | na | nd |
| CD11 | Kilifi | KL 1 | 1997 | 7 | JF451977 | 9 | IL | 1 | JF451871 | 1 |
| CD12 | Kilifi | BR 119 | 1997 | 4 | JF451974 | 3 | II | 1 | JF451872 | 1 |
| CD13 | Kilifi | BR 115 | 1997 | 6 | JF451976 | 4 | II | 1 | JF451873 | 1 |
| CD14 | Kilifi | BR 305 | 1997 | 8 | JF451978 | 5 | IL | 1 | JF451874 | 1 |
| CD15 | Kilifi | BR 135 | 1997 | 1 | JF451950 | 1 | ML | nd | na | nd |
| CD16 | Nyairo^b^ | BT 106 | 2000 | 1 | JF451951 | 1 | ML | nd | na | nd |
| CD17 | Nyairo | IL 57 | 1999 | 3 | JF451972 | 2 | II | nd | na | nd |
| CD18 | Nyairo | BS 224 | 1998 | 1 | JF451952 | 1 | ML | 1 | JF451875 | 1 |
| CD19 | Nyairo | IL 20 | 1999 | 9 | JF451979 | 1 | ML | 2 | JF451881 | 2 |
| CD20 | Nyairo | BS 227 | 1999 | 2 | JF451968 | 2 | II | 4 | JF451892 | 4 |
| CD21 | Nyairo | IL 16 | 1999 | 2 | JF451969 | 2 | II | 1 | JF451876 | 1 |
| CD22 | Nyairo | BR 187 | 1997 | 2 | JF451970 | 2 | II | 4 | JF451893 | 4 |
| CD23 | Nyairo | IL 23 | 1999 | nd | na | nd | na | 2 | JF451882 | 2 |
| CD24 | Nyairo | IL 02 | 1999 | 10 | JF451980 | 7 | ML | 2 | JF451883 | 2 |
| CD25 | Nyairo | BT 108 | 2000 | 11 | JF451981 | 6 | ML | 1 | JF451877 | 1 |
| CD26 | Nyairo | BS 376 | 1999 | 12 | JF451982 | 8 | ML | 1 | JF451878 | 1 |
| CD27 | Nyairo | IL 17 | 1999 | 1 | JF451953 | 1 | ML | 1 | JF451879 | 1 |

^a^ The Kakuzi and Kilifi isolates were obtained during different phases of field trial to determine the efficacy of protection of a recombinant antigen vaccine (p67) against East Coast fever (Musoke et al., 2005).

^b^ The Nyairo isolates were obtained during collaborative research involving National Veterinary Research Centre and ILRI, to study the biological impact of deploying live vaccines against East Coast fever (Odongo et al., 2006).

^δ^ Cloned *T. parva* parasites were derived from a single cell by limiting dilution (Odongo et al., 2006).

**C** Isolates derived from buffalo

| **Sample** | **Animal** | **Year isolated** | **Tp1 alleles** | | | | **Tp2 alleles** | | |
| --- | --- | --- | --- | --- | --- | --- | --- | --- | --- |
|  |  |  | **gene** | **GenBank Acc. No.** | **antigen** | **CTL epitope** | **gene** | **GenBank Acc. No.** | **antigen** |
| BD1 | Mara 3 | 1985 | 16 | JF451995 | 15 | ML | 8 | JF451900 | 8 |
| BD2 | Mara 4 | 1985 | 1 | JF451954 | 1 | ML | 9 | JF451901 | 9 |
| BD3 | Mara 18 | 1985 | 17 | JF451996 | 16 | ML | 10 | JF451902 | 10 |
| BD4 | Mara 30 | 1985 | 1 | JF451955 | 1 | ML | 11 | JF451903 | 11 |
| BD5 | Mara 32 | 1985 | 1 | JF451956 | 1 | ML | 12 | JF451904 | 12 |
| BD6 | Mara 42 | 1985 | 18 | JF451997 | 17 | II | 13 | JF451905 | 13 |
| BD7 | Mara 6998 | 1985 | 1 | JF451957 | 1 | ML | 14 | JF451906 | 14 |
| BD8 | Mara 6999 | 1985 | 19 | JF451998 | 18 | ML | 7 | JF451898 | 7 |
| BD9 | Mara 7001 | 1985 | 20 | JF451999 | 14 | ML | 15 | JF451907 | 15 |
| BD10 | Mara 7546 | 1990 | 21 | JF452000 | 19 | ML | 16 | JF451908 | 16 |
| BD11 | Buffalo 5641 | 1983 | 22 | JF452001 | 14 | ML | 17 | JF451909 | 17 |
| BD12 | Buffalo 6834 | 1984 | 23 | JF452002 | 20 | ML | 18 | JF451910 | 18 |
| BD13 | Buffalo 6837 | 1984 | 14 | JF451990 | 12 | II | 19 | JF451911 | 19 |
| BD14 | Buffalo 7014 | 1987 | 1 | JF451958 | 1 | ML | 20 | JF451912 | 1 |
| BD15 | Buffalo 7698 | 1997 | 14 | JF451991 | 12 | II | 21 | JF451913 | 20 |
| BD16 | Buffalo 7344 | 1989 | 24 | JF452003 | 21 | ML | 7 | JF451899 | 7 |

**D**  Isolates derived from co-cattle grazing with buffalo at Marula farm

| **Sample** | **Animal** | **Vaccine^+^** | **Tp1 alleles** | | | | **Tp2 alleles** | | |
| --- | --- | --- | --- | --- | --- | --- | --- | --- | --- |
|  |  |  | **gene** | **GenBank Acc. No.** | **antigen** | **CTL epitope** | **gene** | **GenBank Acc. No.** | **antigen** |
| BA1 | Marula N6 | FAO1 | 25 | JF452004 | 22 | ML | 22 | JF451914 | 21 |
| BA2 | Marula N13 | control | 26 | JF452005 | 23 | ML | 26 | JF451915 | 22 |
| BA3 | Marula N18 | 3014 | 27 | JF452006 | 14 | ML | 30 | JF451916 | 23 |
| BA4 | Marula N20 | 3014 | 13 | JF451983 | 11 | ML | 24 | JF451917 | 24 |
| BA5 | Marula N33 | FAO1 | 28 | JF452004 | 24 | ML | 25 | JF451918 | 25 |
| BA6 | Marula N36 | 3014 | 13 | JF451984 | 11 | ML | 27 | JF451919 | 26 |
| BA7 | Marula N38 | 3014 | 29 | JF452005 | 25 | ML | 28 | JF451920 | 27 |
| BA8 | Marula N43 | control | 15 | JF451993 | 13 | ML | 31 | JF451921 | 28 |
| BA9 | Marula N50 | control | 30 | JF452006 | 26 | MI | 33 | JF451895 | 6 |
| BA10 | Marula N55 | FAO1 | 14 | JF451992 | 12 | II | 34 | JF451922 | 29 |
| BA11 | Marula N69 | 3014 | 31 | JF452010 | 13 | ML | 35 | JF451923 | 30 |
| BA12 | Marula N76 | control | 13 | JF451985 | 11 | ML | 37 | JF451924 | 31 |
| BA13 | Marula N77 | 3014 | 13 | JF451986 | 11 | ML | 38 | JF451925 | 32 |
| BA14 | Marula N79 | 3014 | 13 | JF451987 | 11 | ML | 40 | JF451926 | 33 |
| BA15 | Marula N86 | 3014 | 32 | JF452011 | 27 | ML | 41 | JF451927 | 34 |
| BA16 | Marula N88 | control | 15 | JF451994 | 13 | ML | 6 | JF451928 | 35 |
| BA17 | Marula N99 | 316 | 13 | JF451988 | 11 | ML | 42 | JF451929 | 36 |
| BA18 | Marula N100 | control | 33 | JF452012 | 28 | ML | 23 | JF451896 | 6 |
| BA19 | Marula N102 | 316 | 1 | JF451959 | 1 | ML | 29 | JF451930 | 1 |
| BA20 | Marula N103 | control | 34 | JF452013 | 29 | MI | 6 | JF451931 | 37 |
| BA21 | Marula N106-1* | 316 | 13 | JF451989 | 11 | ML | 32 | JF451932 | 38 |
| BA22 | Marula N106-2* | 316 |  |  |  | ML | 36 | JF451933 | 39 |
| BA23 | Marula N106-3* | 316 |  |  |  | ML | 6 | JF451897 | 6 |
| BA24 | Marula N106-4* | 316 |  |  |  | ML | 39 | JF451934 | 40 |
| BA25 | Marula N107 | control | 35 | JF452014 | 30 | ML | 43 | JF451935 | 41 |
| **Total** | **82 isolates** |  | **35 alleles** |  | **30 variants** | **4**  **variants** | **43 alleles** |  | **41 variants** |

**Note:** All isolations made during the year 2000.

* Four different Tp2 sequences were obtained from the isolate N106: N106-1, N106-2, N106-3 and N106-4.

^+^ The animals were vaccinated with either the Muguga cocktail (FAO1) or the 3014 or 316 stabilates of *T.parva* Marikebuni.
