# Supplementary material for: Two Theileria parva CD8 T Cell Antigen Genes Are More Variable in Buffalo than Cattle Parasites, but Differ in Pattern of Sequence Diversity
Source: PLoS One. 2011 Apr 29;6(4):e19015. doi: 10.1371/journal.pone.0019015 (PMC3084734; doi:10.1371/journal.pone.0019015)
Supplement: Table S2 — Tp1 gene alleles and their corresponding antigen variants. (DOC) [file pone.0019015.s004.doc]

**Table S2**. Tp1 gene alleles and their corresponding antigen variants.

| **Gene alleles** | **Antigen variants** | **Number of isolates** |
| --- | --- | --- |
| Allele-1 | 1 | 24 |
| Allele-2 | 2 | 11 |
| Allele-3 | 2 | 2 |
| Allele-4 | 3 | 2 |
| Allele-5 | 4 | 1 |
| Allele-6 | 5 | 1 |
| Allele-7 | 6 | 1 |
| Allele-8 | 7 | 1 |
| Allele-9 | 1 | 1 |
| Allele-10 | 8 | 1 |
| Allele-11 | 9 | 1 |
| Allele-12 | 10 | 1 |
| Allele-13 | 11 | 7 |
| Allele-14 | 12 | 3 |
| Allele-15 | 13 | 2 |
| Allele-16 | 14 | 1 |
| Allele-17 | 15 | 1 |
| Allele-18 | 16 | 1 |
| Allele-19 | 17 | 1 |
| Allele-20 | 18 | 1 |
| Allele-21 | 19 | 1 |
| Allele-22 | 18 | 1 |
| Allele-23 | 20 | 1 |
| Allele-24 | 21 | 1 |
| Allele-25 | 22 | 1 |
| Allele-26 | 23 | 1 |
| Allele-27 | 14 | 1 |
| Allele-28 | 24 | 1 |
| Allele-29 | 25 | 1 |
| Allele-30 | 26 | 1 |
| Allele-31 | 13 | 1 |
| Allele-32 | 27 | 1 |
| Allele-33 | 28 | 1 |
| Allele-34 | 29 | 1 |
| Allele-35 | 30 | 1 |
| **Total isolates** | | **79** |
